# Inflammation and DNA methylation coregulate the CtBP-PCAF-c-MYC transcriptional complex to activate the expression of a long non-coding RNA *CASC2* in acute pancreatitis

**DOI:** 10.7150/ijbs.43557

**Published:** 2020-05-18

**Authors:** Jun Zeng, Jian-Yong Chen, Jun Meng, Zhi Chen

**Affiliations:** 1 Department of Gastroenterology, Jiangxi Provincial People's Hospital Affiliated to Nanchang University, Nanchang, Jiangxi, China; 2 Department of critical care medicine, Jiangxi Provincial People's Hospital Affiliated to Nanchang University, Nanchang, Jiangxi, China; 3 Department of Pulmonary and Critical Care Medicine, Tongji Hospital, Tongji University School of Medicine, Shanghai 200065, China.

**Keywords:** acute pancreatitis, CASC2, CtBP, PCAF, c-MYC

## Abstract

Long non-coding RNAs (lncRNAs) are emerging as important regulators involved in the pathogenesis of many diseases. However, it is still unknown if they contribute to the occurrence of acute pancreatitis (AP). Here, we identified a lncRNA *CASC2* (Cancer Susceptibility Candidate 2) was significantly upregulated in the pancreatic tissues from AP patients. Knockdown or overexpression of *CASC2 in vitro* could specifically repress or induce the expression of two proinflammatory cytokines including *IL6* (Interleukin 6) and *IL17*, respectively. Changing the expression levels of several transcription factors that were predicted to bind to the promoter of *CASC2*, we found c-MYC could specifically regulate the expression of *CASC2*. Using immunoprecipitation, mass spectrometry, and co-immunoprecipitation assays, we proved that c-MYC assembled a transcriptional complex with PCAF (p300/CBP-associated Factor) and CtBP1/2 (C-terminal Binding Protein 1 and 2), terming as the CtBP-PCAF-c-MYC (CPM) complex. Further investigation revealed that CtBPs were amplified in the pancreatic tissues from AP patients and they functioned as coactivators to induce the expression of *CASC2* and thus led to the upregulation of *IL6* and *IL17*. Moreover, we identified that decreased DNA methylation levels in the promoters of *CtBPs* and inflammatory stimuli coactivated the expression of *CtBPs.* Collectively, we identified a new signaling pathway in which DNA methylation and inflammatory stimuli coregulate the CPM complex to activate *CASC2* expression, whose induction further activates the expression of *IL6* and *IL17*, eventually aggravating inflammation response and causing the pathology of AP.

## Introduction

Acute pancreatitis (AP) is an inflammatory disorder that occurs in the pancreas and is clinically characterized by severe abdominal pain and increased levels of circulating pancreatic enzymes 1, 2. Current views recognize that gallstones and alcohol are the two major factors that contribute to the occurrence of AP 1, 2. AP is often accompanied by other complications, including low blood pressure, fever, organ damage and pancreatic infection 1, 2. The mortality of AP is still more than 10% even in developed countries 1, 2, which reminds us to pay more attention to investigate the molecular pathogenesis of AP and to identify more therapeutic targets.

Inflammation is a leading cause of AP 3, 4. The destruction of acinar cells in the pancreas can activate macrophages and granulocytes to secrete proinflammatory cytokines, i.e., tumor necrosis factor (TNF)-α, interleukin-1 beta (IL-1β), IL6, IL17 and IL18 5-7. In addition, the activation of multiple signaling pathways has been observed in the pathological process of AP. Of them, the signaling cascades of TLR4/NF-κB (Toll-like receptor 4/Nuclear Transcription Factor-κB) 8, TGF-β/SMAD (Transforming Growth Factor-β/Mothers Against Decapentaplegic Homolog) 9, MAPK (Mitogen-activated Protein Kinase) 10, and JAK/STAT (Janus Kinase/Signal Transducers and Activator of Transcription) pathways have been well characterized 11. Besides, microRNAs (miRNAs) are also involved in the pathogenesis of AP 12. A variety of miRNAs such as miR-7, miR-10, miR-92b, miR-126-5p, miR-551b-5p, miR-24-3p, miR-222-3p, miR-361-5p, and miR-1246 are aberrantly expressed in AP patients 13-18. However, it is still unknown if long non-coding RNAs (lncRNAs) also contribute to the pathogenesis of AP at present. In recent years, emerging evidence indicates that lncRNAs are critical regulators in the process of inflammation response through transcriptionally mediating the expression of inflammatory genes 19. For instance, Zhao and colleagues found that *MALAT1* (Metastasis-associated Lung Adenocarcinoma Transcript 1) could interact with NF-κB to control the expression of *TNFA* and *IL6* 20. Du and colleagues identified that *Mirt2* (Myocardial Infarction-associated Transcript 2) associated with TRAF6 (TNF-associated Factor 6) to prevent the activation of NF-κB and MAPK (Mitogen-Activated Protein Kinase) pathways, thus repressing the expression of proinflammatory cytokines 21. Recently, Zhang and colleagues revealed that *ROCK1* (Regulator of Cytokines and Inflammation) induced by multiple TLR stimuli assembled a ribonucleoprotein complex to control inflammation response 22. Huang and colleagues found that *CASC2* (Cancer Susceptibility 2) was overexpressed in osteoarthritis patients and it could regulate *IL17* expression and contribute to chondrocyte proliferation and apoptosis 23. Except for this unique role in inflammation response, *CASC2* also functions as a tumor suppressor and it is downregulated in multiple cancer types including lung cancer 24, gastric cancer 25, colorectal cancer 26, bladder cancer 27, melanoma 28, and glioma 29. Mechanically, *CASC2* exerts its suppressive role mainly through inhibiting the expression of oncogenic miRNAs and genes. For instance, *CASC2* is able to repress the expression of miR-18a-5p, thereby inducing *PIAS3* (Protein Inhibitor of Activated STAT 3) expression in colorectal cancer cells 26.* CASC2* can interact with miR-181a to control glioma cell growth through upregulating *PTEN* (Phosphatase and Tensin Homolog) pathway 29. In gastric cancer cells, *CASC2* inhibits the phosphorylation levels of ERK1/2 (Extracellular signal-regulated Kinase 1 and 2) and JNK1 (JUN-N-terminal Kinase 1) to decrease cell proliferation 25. In bladder cancer cells, overexpression of *CASC2 in vitro* can decrease the expression of β‐catenin and its downstream target genes *c‐MYC* and *CCND1* (cyclin D1) 27. However, it is still unknown for the mechanism of *CASC2* dysregulation in these biological processes.

Although a large number of lncRNAs have been found to be abnormally expressed in various diseases, the molecular mechanisms that cause their aberrant expression are still poorly understood 16-18. A few studies reveal that DNA methylation and the transcriptional complex can mediate the aberrant expression of lncRNAs 30-32. DNA methylation is a fundamental mechanism that regulates the expression of genes and non-coding RNAs and it occurs in the CpG dinucleotides 33. In mammals, DNA methylation is controlled by three DNA methyltransferases (DNMTs) including DNMT1, DNMT3a and DNMT3b 34. Transcriptional complexes are often composed of transcription factors [e.g., NF-κB, c-MYC, AP1 (Activator Protein 1), and SP1 (Specificity Protein 1)], coactivators [e.g., p300 (E1A Binding Protein 300), CBP (CREB Binding Protein) and PCAF (p300 and CBP-associated Factor)], and corepressors [e.g., CtBPs (C-terminal binding proteins) and RB1 (Retinoblastoma 1)] 35-37. Recently, Zhang and colleagues found that IRF1 (Interferon Regulatory Factor 1) could recruit HDAC1/2 (Histone Deacetylase 1 and 2) and CtBP1 to assemble a complex in the promoter of a lncRNA *GAS5* (Growth Arrest Specific 5) to repress its expression 38.

To identify aberrantly expressed lncRNAs that control the pathogenesis of AP, we carried out a microarray analysis using the pancreatic tissues from AP patients and totally found 21 differentially expressed lncRNAs. We focused the current study on revealing the downstream targets and the upstream signaling of *CASC2*, the mostly upregulated lncRNA in our microarray result. Our results demonstrated that *CASC2* could control the expression of two proinflammatory cytokines including *IL6* and *IL17*. The transcription factor c-MYC recruited PCAF and CtBP1/2 to the promoter of *CASC2* and activated its expression. Both DNA methylation and inflammatory stimuli could trigger the activation of CtBP-associated transcriptional complex, inducing the expression of *CASC2* and its downstream targets.

## Materials and Methods

### Blood sample collection and measurement of serum cytokines

Blood samples were collected from the veins of pancreatic cancer patients (stage 0, setting as controls) (n=48) and AP patients (n=48). The basic information about pancreatic cancer patients and AP patients is summarized in Supplementary Table-1. All participants were aware of the purpose of this study and signed a consent form reviewed and approved by the ethical board of Jiangxi Provincial People's Hospital in China. Blood samples were immediately stored in plastic whole blood tubes with spray-coated K2EDTA (BD, Franklin Lakes, NJ, USA, #367835). The concentrations of cytokines including TNF-α, IL-1β, IL4, IL6, IL8, IL10, IL13, IL15, and IL17 were examined using their corresponding enzyme-linked immunosorbent assay (ELISA) kits purchased from the Thermo Fisher Scientific company (Waltham, MA, USA). The catalog numbers of these kits were as follows: #KHC3011 (TNF-α), #KAC1211 (IL-1β), #KAC1281 (IL4), #KHC0061 (IL6), #KHC0081 (IL8), #KAC1321 (IL10), #BMS231INST (IL13), #BMS2106 (IL15), and #BMS2017 (IL17).

### Pancreatic biopsy collection

Pancreatic biopsies were collected from the same patients as described in blood sample collection using the endoscopic ultrasound-guided fine-needle aspiration (EUS-FNA) method 39. The pancreatic cancer patients had no obvious inflammation in their pancreases according to endoscopic ultrasound results. All participants were aware of the purpose of this study and signed a consent form reviewed and approved by the ethical board of Jiangxi Provincial People's Hospital in China.

### Cell culture and transfection

The human pancreatic epithelial cell line MIA PaCa-2 was obtained from the American Type Culture Collection (ATCC) (Manassas, VA, USA, #CRL-1420) and grown in DMEM (Dulbecco's modified Eagle's medium) (Sigma-Aldrich, #D6046) supplemented with 10% FBS (Fetal Bovine Serum) (Thermo Fisher Scientific, #16000044) and 1% PS (Penicillin-Streptomycin) (Thermo Fisher Scientific, #15140163). Cells under 80% confluence were used for transfections with plasmids or siRNAs, respectively, following a previous protocol 35. The siRNAs used in this study were all purchased from the Thermo Fisher Scientific company and they were included siCASC2-1 (#HSS153939), siCASC2-2 (HSS153940), siCtBP1-1 (#114133), siCtBP1-2 (#2802), siCtBP2-1 (#30207), siCtBP2-2 (#289298), siDNMT1-1 (#S4215), siDNMT1-2 (#S4216), siDNMT3a-1 (#HSS176225), siDNMT3a-2 (#HSS176226), siDNMT3b-1 (#111744), siDNMT3b-2 (#111745), siSP1-1 (#116546), siSP1-2 (#116547), sip50-1 (#107296), sip50-2 (#107296), sip65-1 (#109424), sip65-2 (#116318), sic-JUN-1 (#106741) and sic-JUN-1 (#115273). After transfecting for 48 h, cells were collected and used in the required experiments.

### Protein extraction and western blotting

Three-paired pancreatic biopsies from pancreatic cancer patients (stage 0) and AP patients and cultured cells were used for protein extraction with 1 × RIPA buffer (Sigma-Aldrich, St. Louis, MO, USA, #R0278). Total protein concentrations were determined using a Nanodrop spectrophotometer (Thermo Fisher Scientific, #ND-2000) at 280 nm. Equal amounts of total proteins were loaded onto 12% SDS-PAGE gels for electrophoretic separation. Proteins were then transferred to polyvinylidene difluoride (PVDF) membranes (GE Healthcare, Chicago, IL, USA, #10600023), followed by blocking with 5% milk for 1 h and then probing with primary antibodies including anti-c-MYC (Sigma-Aldrich, #06-340), anti-PCAF (Sigma-Aldrich, #SAB2101734), anti-CtBP1 (BD Biosciences, San Jose, CA, USA, #612042), anti-CtBP2 (BD Biosciences, #612044), anti-Myc (Abcam, Shanghai, China, #ab206486), anti-Flag (Sigma-Aldrich, #SAB4200071), anti-DNMT1 (Sigma-Aldrich, #D4692), anti-DNMT3a (Sigma-Aldrich, #SAB1410305), anti-DNMT3b (Sigma-Aldrich, #SAB2700189), and anti-GAPDH (Abcam, #ab8254). After washing with TBST buffer 5 times, the membrane was further probed with secondary antibodies. Protein signals were recorded with a ChemiDoc Imaging System from Bio-Rad (Hercules, CA, USA).

### Total RNA isolation, microarray and qRT-PCR analyses

The pancreatic biopsies and freshly harvested cells were used for total RNA isolation using the TRIzol^TM^ reagent (Thermo Fisher Scientific, #15596026) according to the manufacturer's instructions. Total RNA concentrations were determined using a Nanodrop spectrophotometer, followed by microarray and qRT-PCR analyses. For microarray analyses, 500 ng RNA of each sample was applied to experiments using an Arraystar Human LncRNA Array V4.0 Chip (Arraystar Inc., #AS-LNC-H-V4.0) and a GeneChip Human Genome U133 Plus 2.0 Array (ThermoFisher Scientific, #900467) following a protocol provided by the manufacturers, respectively. For qRT-PCR analyses, 1.0 μg RNA of each sample was used to synthesize cDNA using a High-Capacity cDNA Reverse Transcription Kit (Thermo Fisher Scientific, #4368814). After diluting 20-fold, cDNAs were subjected to qRT-PCR analyses to detect individual gene expression with primers listed in Supplementary Table-2. The PCR procedures included: 95°C for 3 min, 40 cycles of 95°C for 30 seconds and 68°C for 30 seconds, and 4°C for 10 min. The relative expression of individual genes was normalized to β-Actin using the 2^-ΔΔCt^ method as described previously 35.

### Vector constructions

The coding regions of *CtBP1*, *CtBP2*, *PCAF*, *c-MYC*, *DNMT1*, *DNMT3a*, and *DNMT3b* were amplified by PCR and then cloned into the BamHI and EcoRI sites of pCDNA3-2×Flag and pCDNA3-6×Myc empty vectors, respectively. The coding regions of *SP1*, *p50*, *p65*, and *c-Jun* were cloned into the BamHI and EcoRI sites of pCDNA3-2×Flag empty vectors. The full length of *CASC2* transcript was cloned into the BamHI and EcoRI sites of pCDNA3 empty vector. The resulting vectors were sequenced to verify the correct constructions, followed by purification for transfection. The primers for these constructions were listed in Supplementary Table-3.

### Quantitative methylation-specific PCR (qMSP) analysis

The qMSP analysis was performed following a previous protocol 40. Briefly, the DNA methylation inhibitor AZA (5-Aza-2'-deoxycytidine)-treated cells were subjected to DNA isolation using a PureLink^TM^ Genomic DNA Mini Kit (Thermo Fisher Scientific, #K182001) according to the manufacturer's protocol. After quantification with a Nanodrop spectrophotometer, 1 µg of genomic DNA from each sample was treated with sodium bisulfite using the EZ DNA Methylation-GOLD Kit (Zymo Research, Tustin, CA, USA, #D5006) following a protocol provided by the manufacturer. The resulting genomic DNA was used for qMSP analysis using the TaqMan^®^ Universal Master Mix II non-UNG Kit (Thermo Fisher Scientific, #4440038) with primers listed in Supplementary Table-4.

### Chromatin immunoprecipitation (ChIP) assay

The ChIP assay was performed following a previous protocol 35. Briefly, cells under 80% confluence were crosslinked with 1% formaldehyde for 10 min at room temperature, followed by quenching with glycine at a final concentration of 0.125 M. After fixation, cells were used for immunoprecipitation with a kit (Abcam, #ab185913) according to the method provided by the manufacturer. The antibodies used for immunoprecipitation included anti-CtBP1, anti-CtBP2, anti-PCAF and anti-c-MYC, whose information was the same as descried in the western blotting. The purified input and output DNA were used for qRT-PCR analyses with the same PCR procedure as described in mRNA level detection. The primers were located in the promoter of *CASC2* and their sequences were: forward, 5'-CCGACTTCCCTATGGCTGATGTC-3' and reverse, 5'-GTCCGCGCAGAGGCCTGCACGG-3'.

### Immunohistochemistry (IHC) staining and quantification

Three-paired pancreatic tissues from pancreatic cancer (stage 0) and AP patients were subjected to IHC staining following a previous protocol 41. In brief, the formalin-fixed tissues were sectioned at a thickness of 5 μm, followed by mounting on glass slides. After de-paraffinizing, rehydrating, quenching to remove endogenous peroxidase and antigen retrieval, the slides were incubated with anti-CtBP1 (1:100), anti-CtBP2 (1:100), anti-PCAF (1:200), anti-c-MYC (1:100), anti-IL6 (1:300), anti-IL17 (1:200), anti-DNMT1 (1:100) or anti-DNMT3a (1:100). These antibodies were the same as those used in western blotting assay. After incubating with secondary biotinylated antibodies, the signals of these proteins in tissue sections were visualized using a mouse and rabbit specific HRP/DAB detection kit (Abcam, #ab64264). The quantification of immunohistochemical scores was carried out using the histoscore (H-score) based on the definition and standards described previously.

### Statistical analysis

All experiments were independently performed in triplicate. Statistical analyses of the experimental data were performed using a two-sided Student's t test. The significance levels were set at *P* < 0.05 (*), *P* < 0.01 (**) and *P* < 0.001 (***). The Pearson correlation coefficient (*r*) and associated probability (*P*) were performed to determine the correlation between *CASC2* expression and cytokines, DNMTs and CtBP-associated transcriptional complex members.

## Results

### The concentrations of circulating proinflammatory cytokines were significantly increased in AP patients

**Figure 1:**
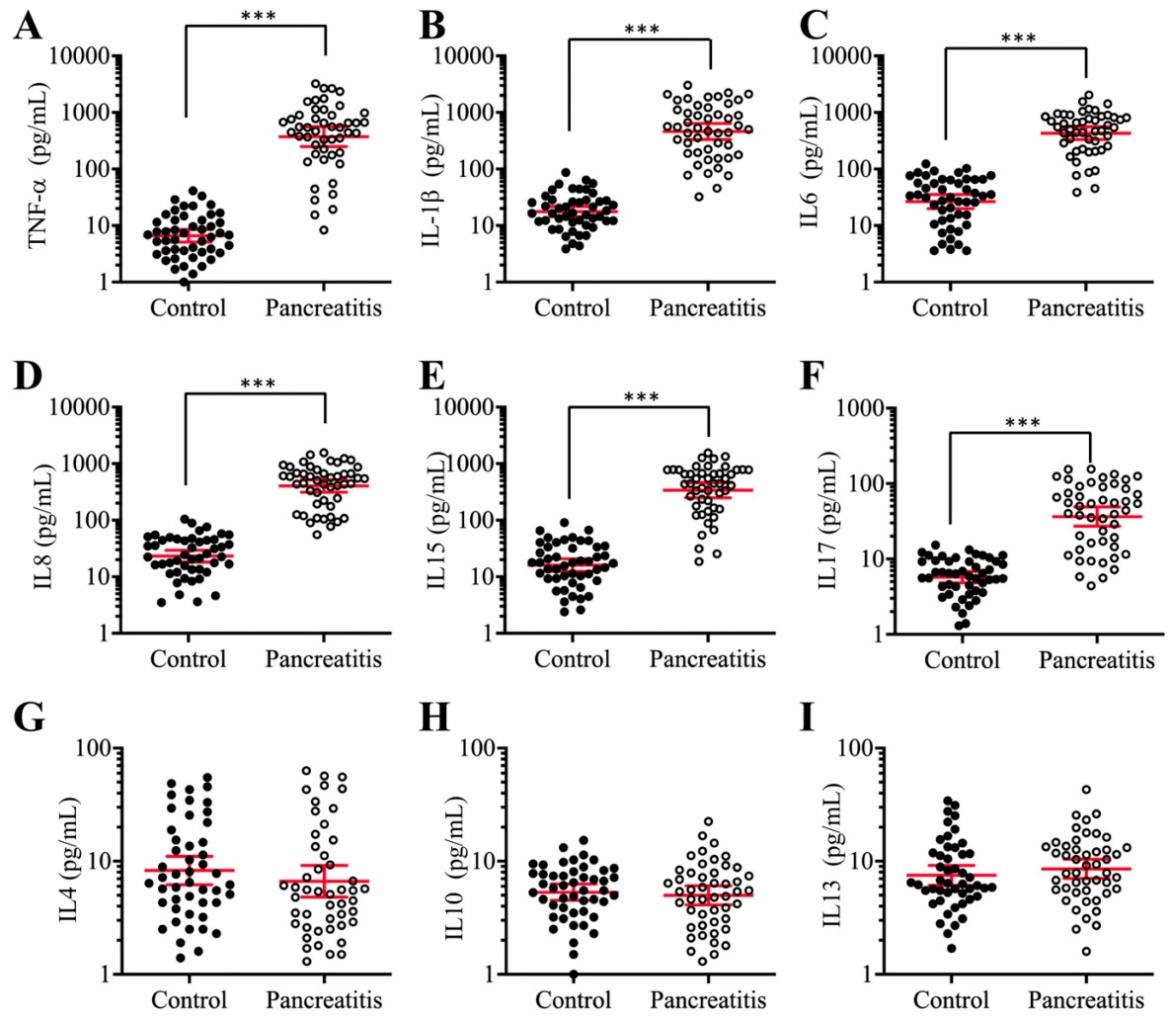
** The serum concentrations of proinflammatory cytokines were significantly increased in AP patients.** Circulating levels of TNF-α **(A)**, IL-1β **(B)**, IL6 **(C)**, IL8 **(D)**, IL15 **(E)**, IL17 **(F)**, IL4 **(G)**, IL10 **(H)**, and IL13 **(I)** were measured in blood samples collected from stage 0 pancreatic patients (control, n = 48) and AP patients (Pancreatitis, n=48) using ELISA assays. *** P < 0.001.

Previous publications have shown increased levels of some proinflammatory cytokines, such as TNF-α, IL-1β and IL8, in pancreatic patients and animal models 42, 43. To verify that the elevated levels of proinflammatory cytokines are a common phenomenon in AP patients, we collected 48-paired blood samples from pancreatic cancer patients (stage 0, control) and AP patients and measured the serum concentrations of six proinflammatory cytokines (TNF-α, IL-1β, IL6, IL8, IL15 and IL17) and three anti-inflammatory cytokines (IL4, IL10 and IL13). The ELISA results indicated that the average levels of all six proinflammatory cytokines in the serum of AP patients were much higher than those in controls (Figures 1A-1F), while the average concentrations of the three anti-inflammatory cytokines were not significantly different between the two groups (Figures 1G-1I). In detail, the concentrations of these cytokines in controls compared to AP patients were as follows: TNF-α (9.23±1.05 pg/mL vs 537.65±78.22 pg/mL, *P* < 0.001) (Figure 1A), IL-1β (19.15±1.69 pg/mL vs 643.69±68.75 pg/mL, *P* < 0.001) (Figure 1B), IL6 (27.55±2.67 pg/mL vs 478.34±37.88 pg/mL, *P* < 0.001) (Figure 1C), IL8 (23.91±2.44 pg/mL vs 311.46±26.57 pg/mL, *P* < 0.001) (Figure 1D), IL15 (17.72±1.64 pg/mL vs 333.88±42.54 pg/mL, *P* < 0.001) (Figure 1E), IL17 (7.22±0.82pg/mL vs 51.43.16±4.25 pg/mL, *P* < 0.001) (Figure 1F), IL4 (8.32±1.13 pg/mL vs 6.87±1.55 pg/mL, *P* = 0.73) (Figure 1G), IL10 (5.09±0.87 pg/mL vs 4.64±0.79 pg/mL, *P* = 0.68) (Figure 1H), and IL13 (7.57±1.22 pg/mL vs 8.46±1.13 pg/mL, *P* = 0.46) (Figure 1I). These results suggested that the elevated levels of proinflammatory cytokines were associated with the occurrence of AP.

### The lncRNA *CASC2* was upregulated in AP patients

**Figure 2:**
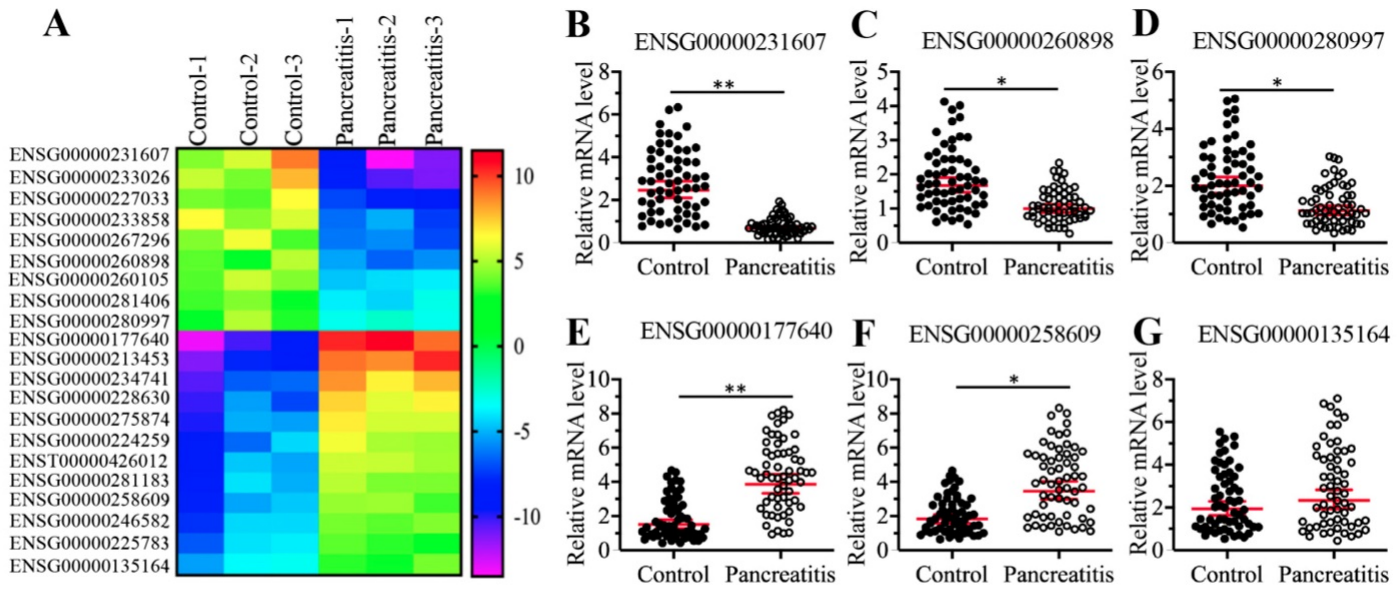
** Identification and verification of differentially expressed lncRNAs in AP patients. (A)** The heatmap of aberrantly expressed lncRNAs. Total RNA from three-paired pancreatic tissues of pancreatic cancer patients (stage 0) (Control-1, -2 and -3) and AP patients (Pancreatitis-1, -2 and -3) were subjected to microarray analysis. The differentially expressed lncRNAs were shown. **(B-G)** Verification of lncRNA expression levels. Total RNA from 48-paired pancreatic tissues of pancreatic cancer patients (Control, stage 0) (Control) and AP patients (Pancreatitis) were subjected to qRT-PCR analyses to measure the expression levels of three downregulated lncRNAs including ENSG00000251562 **(B)**, ENSG00000260898 **(C)**, and ENSG00000280997 **(D)**, and three upregulated lncRNAs including ENSG00000177640 **(E)**, ENSG00000258609 **(F)**, ENSG00000135164 **(G)**. * P < 0.05 and ** P < 0.01.

To identify differentially expressed lncRNAs in the pancreatic tissues, we conducted a microarray analysis using three-paired pancreatic tissues from AP patients and pancreatic cancer patients (stage 0, control). In total, we identified 21 lncRNAs that were consistently downregulated or upregulated in AP patients compared to controls (Supplementary Table-5). Of these differentially expressed lncRNAs, 9 were downregulated, and the remaining 12 were upregulated in AP samples (Figure 2A and Supplementary Table-5). To further evaluate whether the expression of these differentially expressed lncRNAs was associated with AP, we randomly selected three downregulated lncRNAs (ENSG00000251562, ENSG00000260898, and ENSG00000280997) and three upregulated lncRNAs (ENSG00000177640, ENSG00000258609, and ENSG00000135164) and measured their levels in 48-paired pancreatic tissues from the same controls and AP patients as described in blood collection. Consistent with the microarray results, we observed the downregulation of ENSG00000251562, ENSG00000260898 and ENSG00000280997 and the upregulation of ENSG00000177640 and ENSG00000258609 in the 48 AP patients compared to the controls (Figures 2B-2F). However, the expression of ENSG00000135164 was not significantly changed in the same RNA samples (Figure 2G), which suggested that ENSG00000135164 might be a false positive lncRNA in the microarray result. After checking the chromosome loci of these lncRNAs, we found that they were assigned different names and most of them were previously reported to function in the pathogenesis of tumorigenesis (Supplementary Table-5). The most upregulated lncRNA ENSG00000177640 was termed as *CASC2*, which has been characterized as a tumor suppressor in many cancer types 24-29, 44. The significantly aberrant expression pattern of *CASC2* in AP patients inspired us to investigate its role in this process in the following study.

### *CASC2* regulated the expression of *IL6* and *IL17*

**Figure 3:**
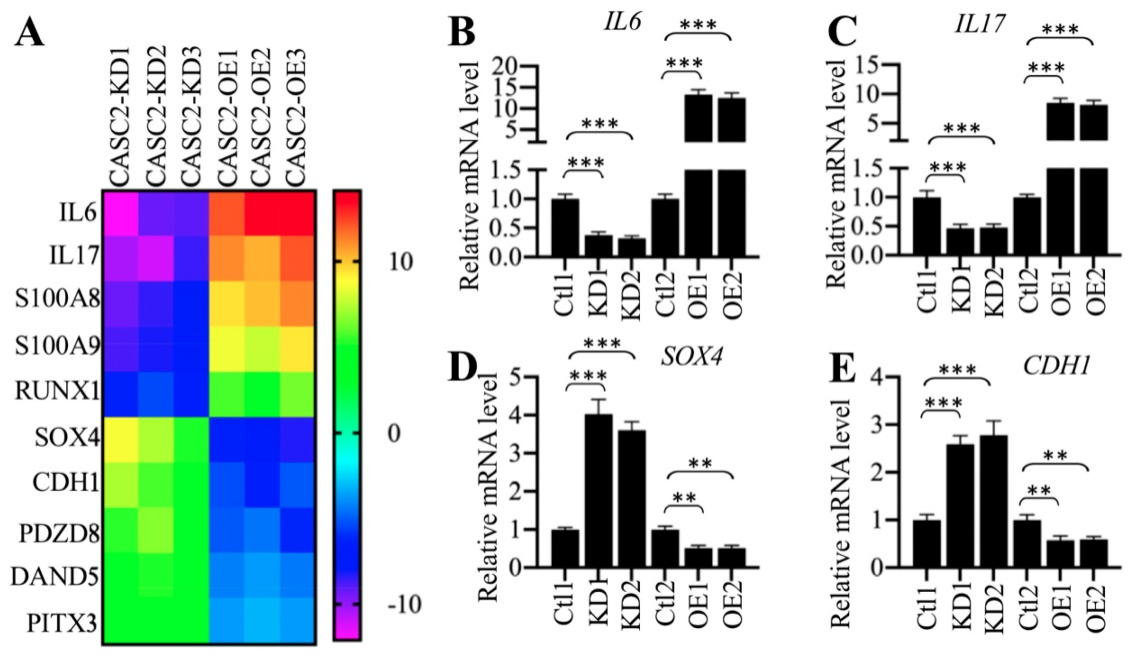
** The expression of *IL6* and *IL17* was dependent on *CASC2.* (A)** Identification of genes dependent on *CASC2*. Total RNA from three independent CASC2-KD (1, 2 and 3) and CASC2-OE (1, 2 and 3) cell lines were subjected to microarray analysis. The top 10 differentially expressed genes were shown. **(B-E)** Verification of gene expression levels. Total RNA from one Control-KD (Ctl1), two independent CASC2-KD cell lines (KD1 and KD2), one Control-OE (Ctl2), and two independent CASC2-OE (OE1 and OE2) were subjected to qRT-PCR analyses to measure the expression levels of *IL6* **(B)**, *IL17* **(C)**, *SOX4* **(D)**, and *CDH1* **(E)**. ** P < 0.01 and *** P < 0.001.

To identify the downstream target genes dependent on *CASC2*, we generated three *CASC2* knockdown (KD) and overexpression (OE) cell lines, respectively, followed by performing a gene-based microarray analysis using these cell lines. Totally, we identified 25 genes that were conversely expressed in three CASC2-KD and CASC2-OE cell lines (Supplementary Table-6). Of these differentially expressed genes, 8 were downregulated in CASC2-KD cells but were upregulated in CASC2-OE cells, while the other 17 were upregulated in CASC2-KD cells but were downregulated in CASC2-OE cells (Supplementary Table-6). Interestingly, we only observed that the changes in *CASC2* expression could affect the expression of *IL6* and *IL17* but not the other proinflammatory cytokine genes (Supplementary Table-6). As shown in Figure 3A, we selected 10 genes that were mostly changed when *CASC2* was knocked down or overexpressed. To further verify our microarray results, we randomly selected four genes including *IL6*, *IL17*, *SOX4* and *CDH1* and determined their dependency on *CASC2*. Consistent with the microarray results, we observed the overexpression of *CASC2* could induce the expression of *IL6* and *IL17* but repress the expression of *SOX4* and *CDH1* (Figures 3B-3F). In contrast, knockdown of *CASC2* resulted in the reverse effects on the expression of these four genes (Figures 3B-3F). Besides, we also measured the concentrations of IL6 and IL17 in the supernatant of cell culture. The ELISA results indicated that both IL6 and IL17 concentrations were decreased in the supernatant of CASC2-KD cells but increased in the supernatant of CASC2-OE cells (Supplementary Figure 1). The dependency of *IL6* and *IL17* on *CASC2* expression *in vitro* together with the results shown in Figures 1C, 1F and 2E suggested that* CASC2* was a key regulator of *IL6* and *IL17* in the pathogenesis of AP. To investigate if the expression of *CASC2* were correlated with circulating *IL6* and *IL17* concentrations in AP patients, we conducted a Pearson correlation assay and found that both of them were positively correlated with the *CASC2* expression (Supplementary Figures 2A and 2B). In contrast, we did not identify a correlation between *IL13* and *CASC2* expression (Supplementary Figure 2C).

### Transcription factor c-MYC specifically regulated the expression of *CASC2*

**Figure 4:**
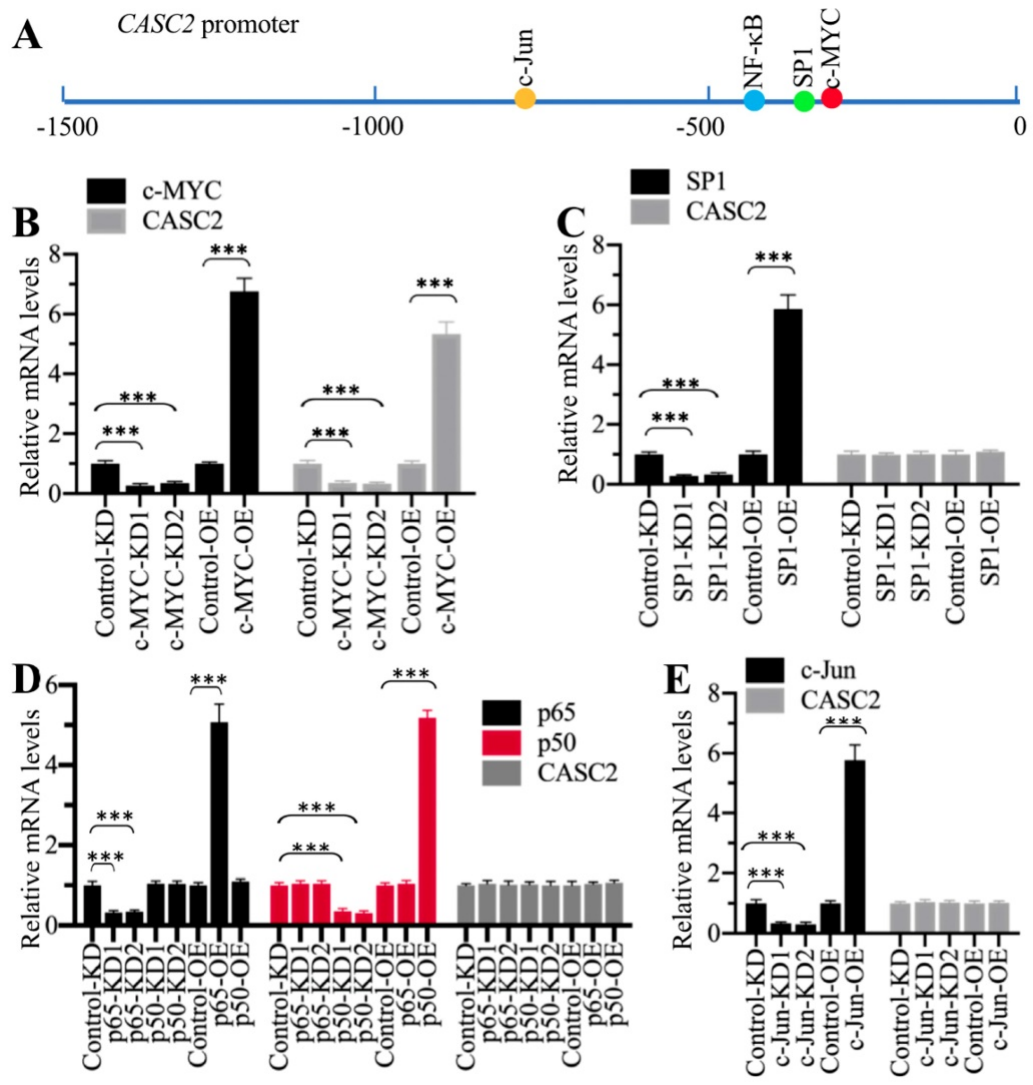
** c-MYC specifically regulated the expression of *CASC2*. (A)** The potential transcription factor binding sites on the promoter of *CASC2*. A 1500-bp length of the *CASC2* promoter was predicted the transcription factor binding sites. One c-MYC, one SP1, one NF-κB and one c-JUN binding sites were found, and their positions were shown. **(B)** Knockdown or overexpression of *c-MYC* changed the expression of *CASC2*. Total RNA from Control-KD, c-MYC-KD1, c-MYC-KD2, Control-OE, and c-MYC-OE cells were applied to qRT-PCR analyses to measure the mRNA levels of *c-MYC* and *CASC2*. ***P<0.001. **(C)** Knockdown or overexpression of *SP1* could not change the expression of *CASC2*. Total RNA from Control-KD, SP1-KD1, SP1-KD2, Control-OE, and SP1-OE cells were applied to qRT-PCR analyses to measure the mRNA levels of *SP1* and *CASC2*. ***P<0.001. **(D)** Knockdown or overexpression of *NF-κB* subunits could not change the expression of *CASC2*. Total RNA from Control-KD, p50-KD1, p50-KD2, p65-KD1, p65-KD2, Control-OE, p50-OE and p65-OE cells were applied to qRT-PCR analyses to measure the mRNA levels of *p50*,* p65* and *CASC2*. ***P<0.001.** (E)** Knockdown or overexpression of *c-JUN* could not change the expression of *CASC2*. Total RNA from Control-KD, c-JUN-KD1, c-JUN-KD2, Control-OE, and c-JUN-OE cells were applied to qRT-PCR analyses to measure the mRNA levels of *c-JUN* and *CASC2*. ***P<0.001.

To reveal the mechanism underlying *CASC2* overexpression in AP patients, we initially analyzed its promoter to determine if transcription factors could control the expression of *CASC2*. We selected a length of 1500-bp promoter region of *CASC2* and scanned the potential transcription factor binding sites. As shown in Figure 4A, we found four conserved transcription factor binding sites including c-MYC [-356-(-361), CACGTG], SP1 [-390-(-399), CCCCCGCCCC], NF-κB [-414-(-423), GGGGGTCCCC], and c-JUN [-762-(-768), TGTGTCA] using their consensus sequences. We individually knocked down and overexpressed these transcription factors. After verifying their successful downregulation and upregulation with immunoblots (Supplementary Figure 3), we evaluated their effects on *CASC2* expression. As shown in Figure 4B, knockdown or overexpression of *c-MYC* decreased or increased *CASC2* level, respectively. However, knockdown or overexpression of the other three transcription factors could not change the expression of *CASC2* (Figures 4C-4E). These results suggested that c-MYC could specifically regulate the expression of *CASC2* at the transcriptional level.

### c-MYC recruited PCAF and CtBPs to assemble a transcriptional complex

**Figure 5:**
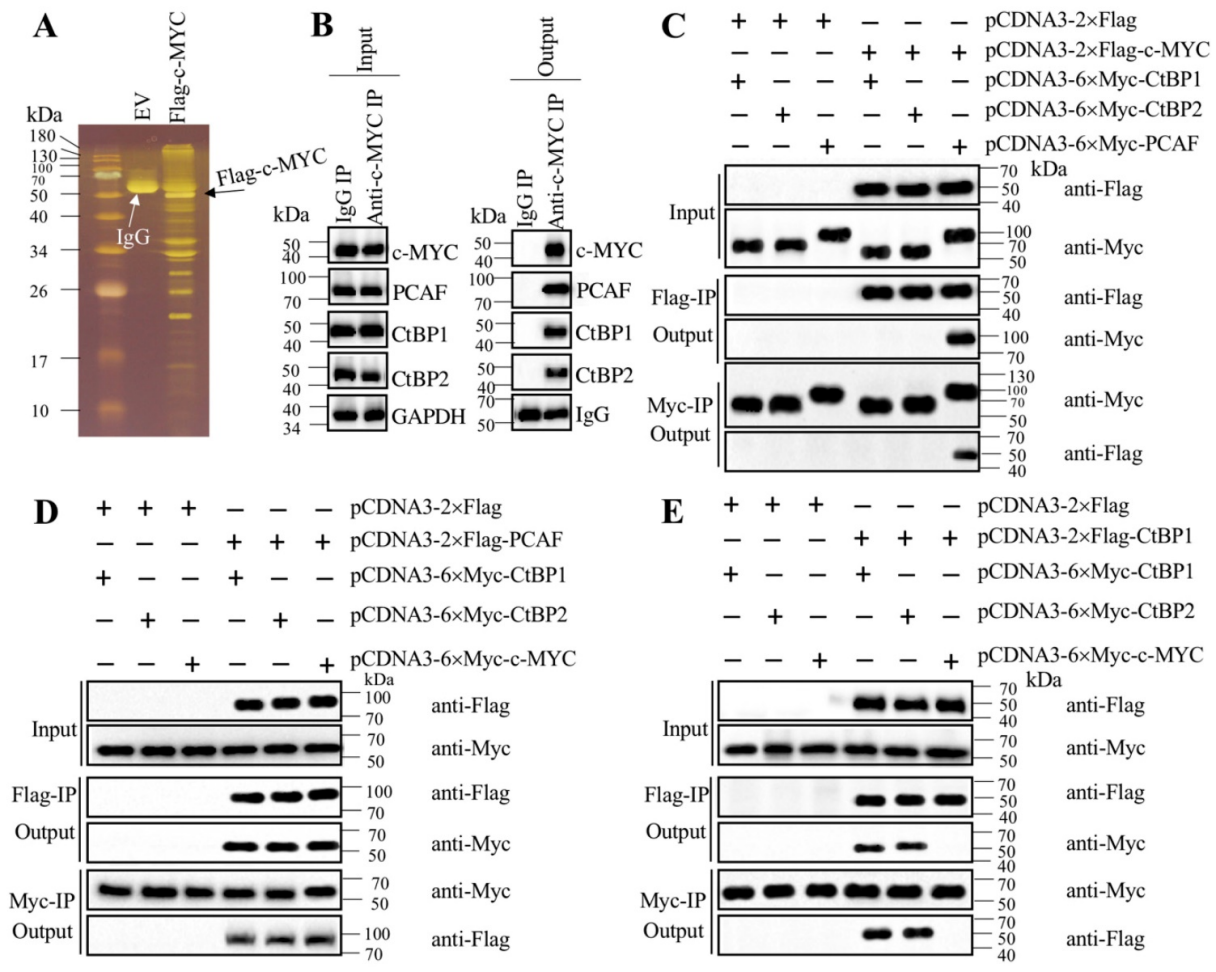
** c-MYC associated with PCAF and CtBPs to assemble a complex. (A)** The Flag-c-MYC-associated complex. The pCDNA3-2×Flag (empty vector, EV) and pCDNA3-2×Flag-c-MYC plasmids were transfected into MIA PaCa-2 cells, respectively. The resulting cells were subjected to immunoprecipitation with the anti-Flag resin. The purified complexes were separated in an SDS-PAGE gel and incubated with a silver staining kit. The IgG and Flag-c-MYC were indicated by arrows. **(B)** c-MYC could pull down PCAF and CtBPs *in vivo*. Equal weight of pancreatic tissues from three AP patients was mixed and lysed, and 1/11 cell extracts were used as an input, and the other 10/11 cell extracts were equally divided into two parts, followed by immunoprecipitation with an IgG and anti-c-MYC antibody-associated protein A beads, respectively. The input and output proteins were used to determine protein levels of c-MYC, PCAF, CtBP1 and CtBP2, respectively. **(C)** c-MYC directly interacted with PCAF but not CtBPs *in vitro*. The MIA PaCa-2 cells were transfected with different plasmids including pCDNA3-2×Flag + pCDNA3-6×Myc-CtBP1, pCDNA3-2×Flag + pCDNA3-6×Myc-CtBP2, pCDNA3-2×Flag + pCDNA3-6×Myc-PCAF, pCDNA3-2×Flag-c-MYC + pCDNA3-6×Myc-CtBP1, pCDNA3-2×Flag-c-MYC + pCDNA3-6×Myc-CtBP2, and pCDNA3-2×Flag-c-MYC + pCDNA3-6×Myc-PCAF. The resulting cells were lysed and immunoprecipitated with an anti-Flag and anti-Myc resins, respectively, followed by immunoblots to examine the input and output proteins levels using anti-Flag and anti-Myc antibodies.** (D)** PCAF directly interacted with both c-MYC and CtBPs *in vitro*. The MIA PaCa-2 cells were transfected with different plasmids including pCDNA3-2×Flag + pCDNA3-6×Myc-CtBP1, pCDNA3-2×Flag + pCDNA3-6×Myc-CtBP2, pCDNA3-2×Flag + pCDNA3-6×Myc-c-MYC, pCDNA3-2×Flag-PCAF + pCDNA3-6×Myc-CtBP1, pCDNA3-2×Flag-PCAF + pCDNA3-6×Myc-CtBP2, and pCDNA3-2×Flag-PCAF + pCDNA3-6×Myc-c-MYC. The resulting cells were lysed and immunoprecipitated with an anti-Flag and anti-Myc resins, respectively, followed by immunoblots to examine the input and output protein levels using anti-Flag and anti-Myc antibodies.** (E)** CtBPs assembled a heterotetramer *in vitro*. The MIA PaCa-2 cells were transfected with different plasmids including pCDNA3-2×Flag + pCDNA3-6×Myc-CtBP1, pCDNA3-2×Flag + pCDNA3-6×Myc-CtBP2, pCDNA3-2×Flag + pCDNA3-6×Myc-c-MYC, pCDNA3-2×Flag-CtBP1 + pCDNA3-6×Myc-CtBP1, pCDNA3-2×Flag-CtBP1 + pCDNA3-6×Myc-CtBP2, and pCDNA3-2×Flag-CtBP1 + pCDNA3-6×Myc-c-MYC. The resulting cells were lysed and immunoprecipitated with an anti-Flag and anti-Myc resins, respectively, followed by immunoblots to examine the input and output proteins levels using anti-Flag and anti-Myc antibodies.

Transcription factors often recruit coactivators and corepressors to assemble transcriptional complexes 35-37. To investigate how c-MYC activated *CASC2* in the pathogenesis of AP, we performed immunoprecipitation in cells expressing pCDNA3-2×Flag (empty vector, EV) and pCDNA3-2×Flag-c-MYC (Figure 5A). The purified Flag-c-MYC complex was applied to mass spectrometry analysis to identify c-MYC-associated proteins. Totally, we obtained 35 proteins in this complex (Supplementary Table-7). After analyzing the candidate protein list, we found a histone acetyltransferase PCAF and two corepressors CtBP1 and CtBP2, which are well-known components of a transcriptional machinery. To determine if c-MYC could associate these three proteins to assemble a complex *in vivo*, we performed an immunoprecipitation assay in AP tissues using anti-c-MYC antibody. The purified protein complex was subjected to immunoblots to examine the existence of PCAF and CtBP1/2. The western blotting results indicated that c-MYC could pull down PCAF and CtBP1/2 together (Figure 5B), which suggested that c-MYC could assemble a complex with these three proteins *in vivo*. To further determine how this complex was assembled, we performed coimmunoprecipitation (Co-IP) assay to examine the direct interactions between c-MYC and PCAF, c-MYC and CtBPs, PCAF and CtBPs, as well as CtBP1 and CtBP2. The Co-IP results indicated that c-MYC could interact with PCAF directly but not with CtBPs (Figure 5C), and PCAF could directly interact with both c-MYC and CtBPs (Figures 5C and 5D). In addition, CtBP1 and CtBP2 could interact with themselves and each other (Figure 5E), suggesting that they could form a heterotetramer. These results clearly suggested that c-MYC directly recruited PCAF, which acted as a linker to bind to the CtBP heterotetramer to assemble the CtBPs-PCAF-c-MYC (CPM) complex. To determine if this complex specifically bond to the promoter of *CASC2*, we conducted ChIP assays in c-MYC-KD and c-MYC-OE cells to evaluate the occupancies of CPM components. The ChIP results showed that knockdown of *c-MYC* significantly decreased the enrichment of CPM components in the promoter of *CASC2* (Supplementary Figure 4A), while overexpression of *c-MYC* increased their occupancies (Supplementary Figure 4B). These results suggested that the CPM complex was required for the regulation of *CASC2* overexpression in the pathogenesis of AP.

### CtBPs functioned as coactivators to regulate the expression of *CSAC2*

**Figure 6:**
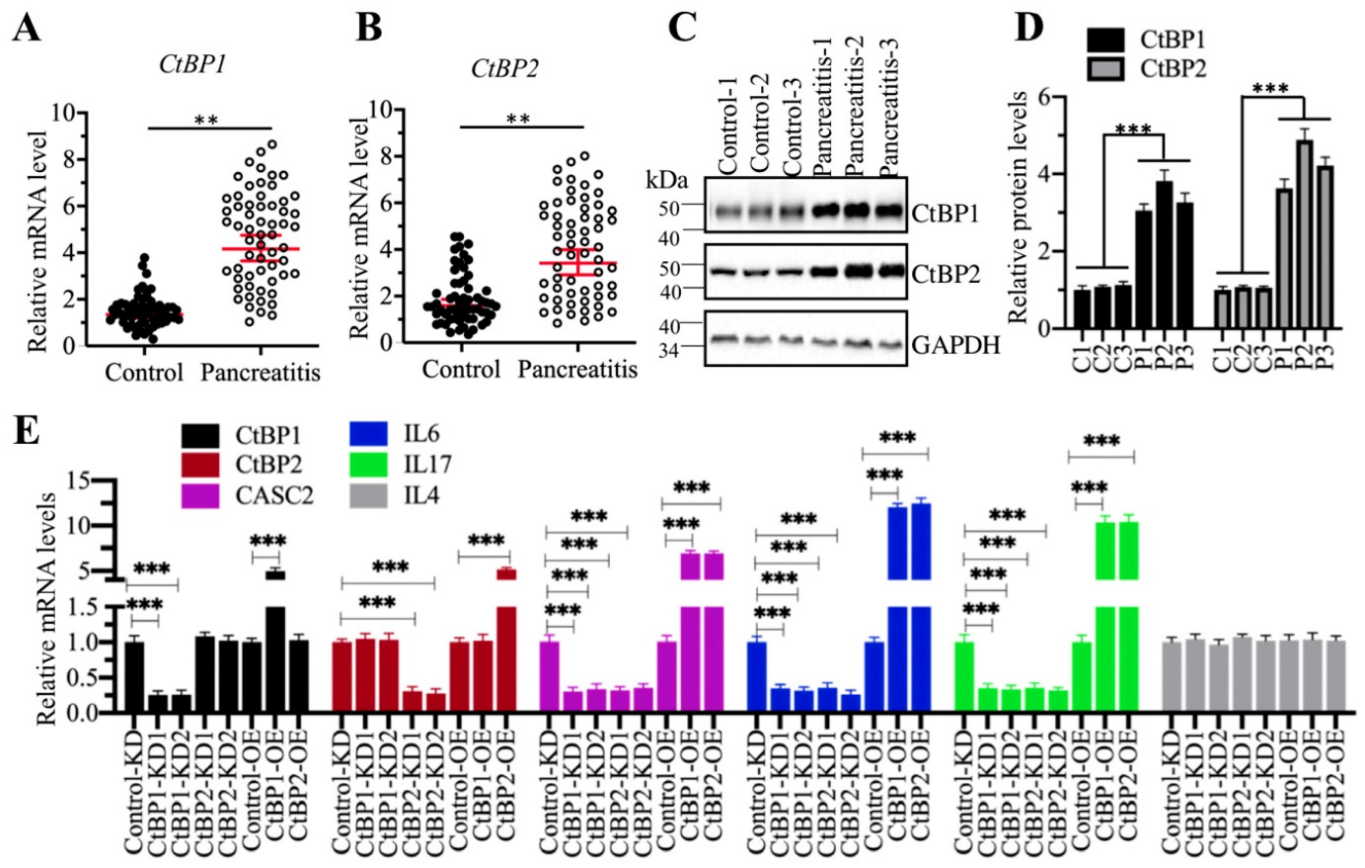
***CtBPs* were overexpressed in AP patients. (A** and** B)** *CtBPs* were overexpressed in AP patients. Total RNA from 48-paired pancreatic tissues of pancreatic cancer patients (Control, stage 0) and AP patients (Pancreatitis) were subjected to qRT-PCR analyses to measure the mRNA levels of *CtBP1* **(A)** and *CtBP2* **(B)**. **P < 0.01. **(C)** CtBP protein levels were elevated in AP patients. Three-paired pancreatic tissues from controls (1, 2 and 3) and AP patients (1, 2 and 3) were used to determine protein levels of CtBP1 and CtBP2. GAPDH was probed as a loading control. **(D)** The relative protein levels of CtBPs. The protein bands in (C) were quantified and normalized to GAPDH. ***P < 0.001. **(E)** The effects of knockdown or overexpression of *CtBPs* on the expression of *CASC2*, *IL6*, *IL17* and *IL4*. Total RNA from Control-KD, CtBP1-KD1, CtBP1-KD2, CtBP2-KD1, CtBP2-KD2, Control-OE, CtBP1-OE and CtBP2-OE were used to examine mRNA levels of *CtBP1*, *CtBP2*, *CASC2*, *IL6*, *IL17* and *IL4* by qRT-PCR analyses. ***P < 0.001.

CtBPs can function as either corepressors or coactivators in different biological processes 35-37. To determine how CtBPs functioned in the regulation of *CASC2* expression, we primarily detected the mRNA and protein levels of *CtBPs* in the pancreatic samples from AP and control patients. The qRT-PCR results showed that both *CtBP1* and *CtBP2* mRNA levels were significantly upregulated in comparison to controls (Figures 6A and 6B). We then randomly selected three-paired tissues as represent to detect the protein levels CtBP1 and CtBP2. Similarly, we also observed the accumulation of CtBP protein levels in AP samples compared to controls (Figures 6C and 6D). The similar expression patterns of *CtBPs* with *CASC2* implied that they might transactivate *CASC2* expression. To verify this hypothesis, we created CtBP-KD and CtBP-OE cell lines and then examined the expression levels of *CASC2* and its downstream targets *IL6* and *IL17* in these cells. As expected, we observed that knockdown of *CtBPs* repressed the expression of *CASC2*,* IL6* and *IL17* while overexpression of *CtBPs* caused the reverse effects on the expression of *CASC2* and its targets (Figure 6E). In addition, we also conducted a Pearson correlation assay to determine the correlation between *CtBPs* and *CASC2* in pancreatic samples from AP patients. As shown in Supplementary Figure 5, the expression of both *CtBP1* and *CtBP2* was positively correlated with *CASC2*. These results together supported the conclusion that CtBPs acted as coactivators instead of corepressor in the regulation of *CASC2* expression.

### Decreased DNA methylation levels in the promoters of *CtBPs* contributed to their overexpression in AP pancreatic tissues

**Figure 7:**
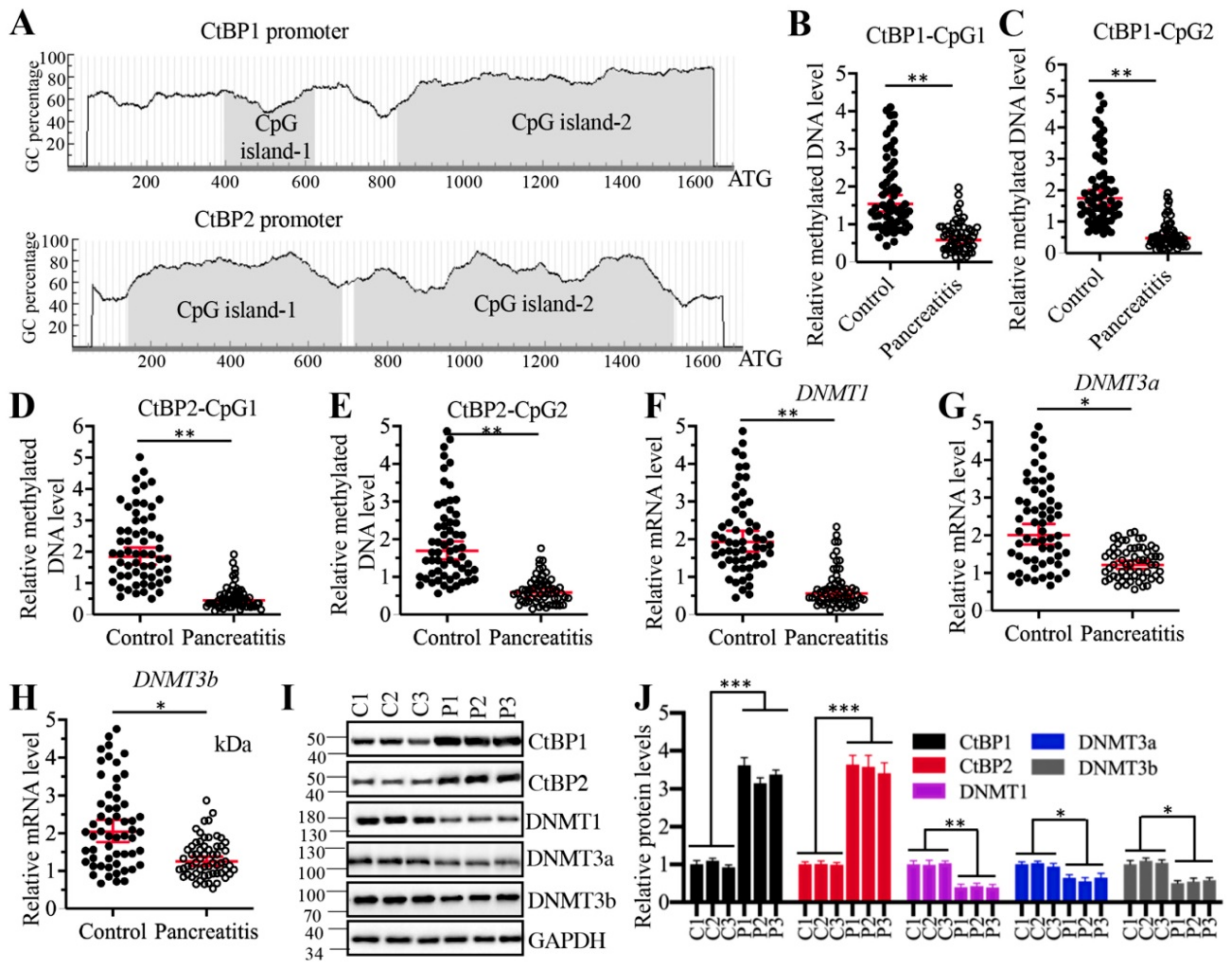
** Decreased DNA methylation levels in the promoters of *CtBP*s were responsible for their overexpression. (A)** Both *CtBP1* and* CtBP2* promoters had two CpG islands. A 1700-bp length of *CtBP* promoters was selected to predict the CpG island and two CpG islands were identified in each gene.** (B**-**E)** The relative DNA methylation levels of CpG islands in AP patients. Pancreatic tissues from 48-paired controls and AP patients were subjected to isolate genomic DNA, followed by treating with sodium bisulfite. The qMSP analyses were performed to examine the methylated DNA levels in the CpG islands of *CtBP1* **(B** and **C)** and *CtBP2* **(D** and** E)** promoters. ** P < 0.01. **(F-H)** DNMTs were downregulated in AP patients. Total RNA from 48-paired pancreatic tissues of controls and AP patients were subjected to qRT-PCR analyses to measure the mRNA levels of *DNMT1* **(F)**, *DNMT3a* **(G)** and *DNMT3b* **(H)**. *P < 0.05 and **P < 0.01. **(I)** DNMT protein levels were decreased in AP patients. Three-paired pancreatic tissues from controls (1, 2 and 3) and AP patients (1, 2 and 3) were used to determine protein levels of CtBP1, CtBP2, DNMT1, DNMT3a and DNMT3b, respectively. GAPDH was probed as a loading control. **(J)** The relative protein levels of DNMTs. The protein bands in (I) were quantified and normalized to GAPDH. *P < 0.05, **P < 0.01 and ***P < 0.001.

Given that *CtBPs* were upregulated at the transcriptional level, we next aimed to investigate the underlying mechanisms of their overexpression. DNA methylation is a fundamental mechanism that regulates gene expression. To investigate if DNA methylation was involved in the regulation of *CtBP* overexpression, we selected a 1700-bp length of *CtBP* promoters and predicted if they were abundant in GC nucleotides in a website (http://www.urogene.org). As shown in Figure 7A, both *CtBP1* and *CtBP2* promoters were abundant in GC nucleotides and they had two CpG islands. We then examined the methylated levels in the CpG islands. Surprisingly, we observed that the methylated levels of CpG islands in both *CtBP1* and *CtBP2* promoters were significantly decreased in AP pancreatic samples compared to controls (Figures 7B-7E). These results implied that the expression of *DNMTs* might be changed in AP tissues. To validate this hypothesis, we examined the mRNA and protein levels of three *DNMTs* including *DNMT1*, *DNMT3a* and *DNMT3b* in three-paired pancreatic tissues from AP patients and controls. The results indicated that all three *DNMTs* were downregulated in their mRNA and protein levels in AP patients (Figure 7F-7J). To determine if changes of *DNMT* levels affected the expression of *CtBPs*, we generated two independent knockdown and one overexpression cell lines of each *DNMT* genes. After confirming their successful knockdown and overexpression (Supplementary Figures 6A and 6B), we measured *CtBP* levels in these cells. The qRT-PCR results indicated that knockdown of *DNMTs* caused the upregulation of *CtBPs* while overexpression of *DNMTs* resulted in the downregulation of *CtBPs* (Supplementary Figures 6A and 6B). We also observed similar patterns of CtBP protein levels in DNMT-KD and DNMT-OE cells (Supplementary Figures 6C and 6D). These results suggested that decreased DNA methylation levels in the *CtBP* promoters were responsible for their overexpression. In addition, we also measured the mRNA levels of *CASC2*, *IL6* and *IL17* in these DNMT-KD and DNMT-OE cells. Consistent with the expression of *CtBPs* in these cells, we also observed the increase or decrease of *CASC2*, *IL6* and *IL17* levels in DNMT-KD or DNMT-OE cells, respectively (Supplementary 7). To determine if the expression of *DNMTs* was correlated with *CASC2*, we also conducted a Pearson correlation assay and found *DNMT* mRNA levels were negatively correlated with *CASC2* expression (Supplementary Figure 8). Thus, we revealed a mechanism in which the downregulation of DNMTs caused the induction of *CtBPs*, which further assembled a CPM complex to activate *CASC2* expression. To further support this conclusion, we also carried out IHC staining assay to determine the protein levels of DNMTs, CtBPs, PCAF, c-MYC and two proinflammatory cytokines IL6 and IL17 in the pancreatic tissues from AP patients and controls. The IHC staining results of these proteins and their corresponding quantification results also showed CtBPs, PCAF, c-MYC, IL6 and IL17 protein levels were much higher while DNMT protein levels were much lower in AP patients compared to controls (Supplementary Figure 9).

### Inflammation stimuli induced the expression of *CtBPs*

**Figure 8:**
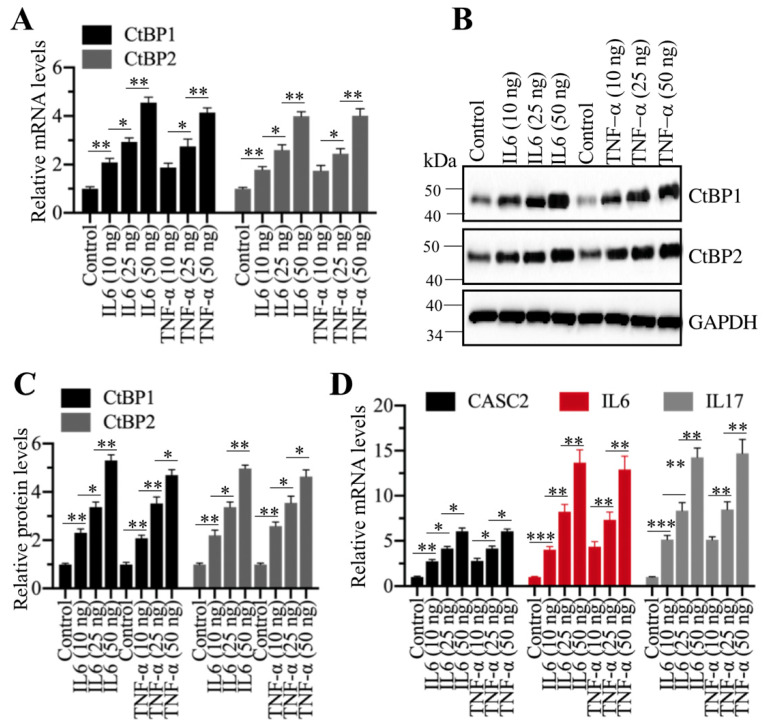
** Recombinant IL6 and TNF-α induced the expression of *CtBPs* at both transcriptional and protein levels. (A)** IL6 and TNF-α induced the mRNA levels of *CtBPs**.*** The MIA PaCa-2 cells were treated with IL6 and TNF-α at the concentrations of 0, 10, 25 and 50 ng/mL for 6 h. The resulting cells were used for RNA isolation, followed by qRT-PCR analyses to determine the mRNA levels of *CtBP1* and *CtBP2*. **P<0.01 and ***P<0.001.** (B)** IL6- and TNF-α induced the protein levels of CtBPs. Cells used in (A) were subjected to immunoblots to examine protein levels of CtBP1 and CtBP2. GAPDH was probed as a loading control. **(C)** The relative protein levels of CtBPs. The protein bands in (B) were quantified and normalized to GAPDH. **P < 0.01 and ***P < 0.001. **(D)** IL6 and TNF-α induced the mRNA levels of *CASC2*,* IL6* and* IL17*. RNA samples used in (A) were subjected to examine mRNA levels of* CASC2*,* IL6* and* IL17* by qRT-PCR analyses. **P < 0.01 and ***P < 0.001.

Given that *CtBPs* were significantly amplified in AP tissues, we were curious if inflammation stimuli could induce their expression. For this purpose, we treated cells with different concentrations (0, 10, 25 and 50 ng/mL) of recombinant IL6 or TNF-α, followed by examining *CtBP* mRNA and protein levels. Interestingly, the qRT-PCR results showed that both IL6 and TNF-α treatments could induce the expression of *CtBPs* in a dose-dependent manner (Figure 8A). Similarly, we also observed a dose-dependent induction of CtBP protein levels in IL6 or TNF-α-treated cells (Figures 8B and 8C). Moreover, we also measured the mRNA levels of *CASC2*, *IL6* and *IL17* in IL6- or TNF-α-treated cells. Consistent with *CtBP* expression patterns, we also found a dose-dependent induction of *CASC2*, *IL6* and *IL17* (Figure 8D). These results suggested that inflammation microenvironment in AP patients might also contribute to the overexpression of *CtBPs*.

### DNA methylation and inflammation stimuli coordinately regulated the expression of *CtBPs*

Our above results indicated that both DNA methylation and inflammation stimuli could regulate the expression of *CtBPs*. To determine if DNA methylation and inflammation stimuli could function coordinately, we treated Control-KD and DNMT1-KD cells with 50 ng/mL recombinant IL6 and TNF-α, respectively, followed by measuring mRNA levels of *CtBPs*. The qRT-PCR results indicated that *CtBP* mRNA levels were significantly upregulated in IL6- or TNF-α-treated DNMT1-KD cells in comparison to untreated DNMT1-KD cells or in IL6- or TNF-α-treated Control-KD cells (Supplementary Figure 10A). We also observed similar patterns of CtBP protein levels (Supplementary Figures 10B and 10C). In addition, we also found that the expression of *CASC2*, *IL6* and *IL17* was significantly upregulated in IL6- or TNF-α-treated DNMT1-KD cells compared to DNMT1-KD cells or IL6- or TNF-α-treated Control-KD cells (Supplementary Figure 10D). These results demonstrated that the decreased DNA methylation levels and inflammation stimuli could coordinately regulate the expression of *CtBPs* and *CASC2*. To determine if knockdown of *DNMT1* and inflammation stimuli affected the occupancies of CPM members on the promoter of *CASC2*, we carried out ChIP assays using antibodies against CPM members. The qRT-PCR results revealed that either knockdown of *DNMT1* or IL6/TNF-α treatment could significantly increase the occupancies of CPM members on the promoter of *CASC2*, and this increase could be further enhanced in DNMT1-KD cells treated with The IL6 or TNF-α (Supplementary Figure 11).

## Discussion

**Figure 9:**
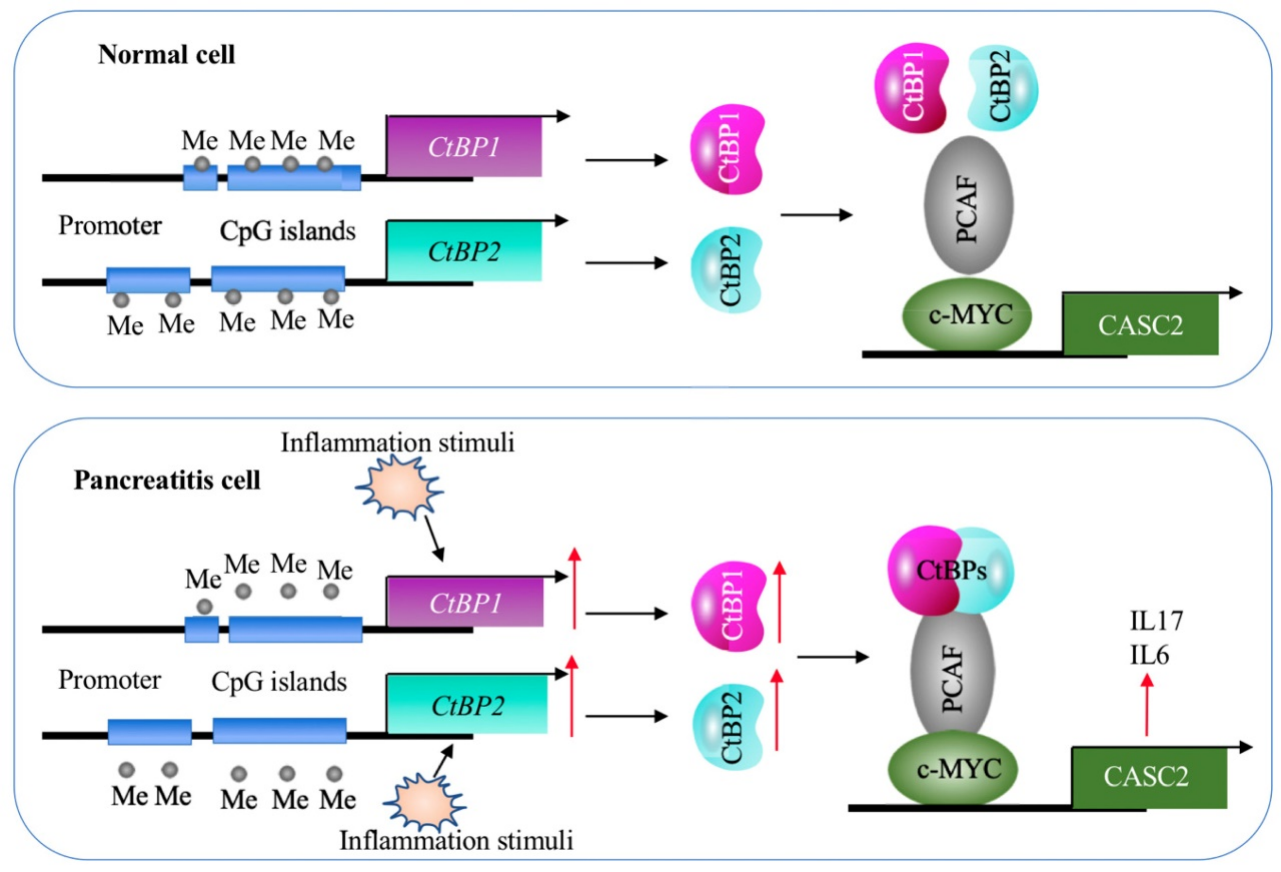
** A schematic model of *CASC2* in normal cells and pancreatitis cells.** In normal cells, the DNA methylation levels in the promoters of *CtBPs* are very low and there are no intracellular and environmental inflammation stimuli. Thus, the expression of *CtBPs* is maintained at a basal level. As a result, CtBPs proteins cannot efficiently associate with PCAF and c-MYC to assemble a complex to activate the expression of *CASC2* and its targets *IL6* and *IL17*. In pancreatitis cells, the decreased DNA methylation levels in the promoters of *CtBPs* and environmental inflammation stimuli activate the expression of *CtBPs*. The amplified CtBPs form a heterotetramer, which is then recruited by the c-MYC-PCAF complex to assemble the CPM transcriptional machinery. The activation of CPM complex specifically binds to the promoter *CASC2* to induce its expression, further leading to the induction of *IL6* and *IL17*.

Although lncRNAs are involved in the pathogenesis of many diseases, it is still unknown their roles in AP. In the present study, we identified a lncRNA *CASC2* was significantly upregulated in the pancreatic tissues of AP patients. Screening genes dependent on *CASC2*, we found that two proinflammatory cytokine genes *IL6* and *IL17* were positively regulated by *CASC2*. We then revealed a transactivation mechanism in which the decreased DNA methylation levels and inflammation stimuli coactivated the expression of *CtBPs*, and the increased CtBPs associated with PCAF and c-MYC to assemble the CPM complex, binding to the promoter of *CASC2* and activating its expression (Figure 9). The clear description of this new signaling pathway will provide more potential targets for developing therapeutic strategies to prevent AP progression.

CtBP1 and CtBP2 are two conserved proteins with more than 80% amino acid sequence identify 35-37. However, these two proteins do not show obvious redundancy in their functions 35-37. Both of them are separately amplified in different cancers. In the tumorigenesis, CtBPs function as corepressors and they can cooperate with transcription factors to repress the expression of tumor suppressors such as *Bax* (BCL2 Associated X protein), *Bim* (also known as *BCL2L11*, BCL2 Like 11), *BRCA1* (Breast Cancer Type 1), and *CDH1* (Cadherin 1) 37. Moreover, CtBPs also have transactivation roles. For example, CtBP2 can activate *Tiam1* (T cell lymphoma Invasion and Metastasis 1) in an NADH-dependent manner, thereby promoting cancer cell migration 45. CtBP2 physically interacts with TCF4 (Transcription factor 4) and activates TCF4-mediated signaling to regulate cancer stem cell growth and self-renewal 46. CtBP1 can transcriptionally activate the expression of *MDR* (Multidrug resistance) gene, thereby contributing to chemotherapeutic drug resistance 47. CtBP2 assembles a complex with p300 and AP1 to activate the expression of *TGFB*, causing chronic renal failure 48. Except for regulating gene expression, CtBP-coupled transcriptional complexes have also been found to regulate the expression of miRNAs and lncRNAs. For instance, CtBP2 associates with HDAC1 and FOXP3 (Forkhead Box P3) to assemble a complex to repress miR-199a-3p, thereby attenuating its inhibition of *NLRP1* (NLR Family Pyrin Domain Containing 1) and causing acute lung injury 35. CtBP1 couples with HDAC1/2 and IRF1 to repress the expression of *GAS5* in human osteosarcoma 38. Interestingly, our current study revealed that both decreased DNA methylation levels in the promoters of *CtBPs* and inflammation stimuli contribute to the overexpression of *CtBPs*. To our knowledge, our results for the first time found this interesting regulatory effect. These results demonstrate that the diverse roles of CtBPs in different biological processes. Several CtBP inhibitors such as MTOB (2-Keto-4-butyric acid), HIPP (2-Hydroxyimino-3-phenyl-propionic acid), CP61 and NSC95397 have been reported to disrupt CtBP-mediated signaling 49. Thus, it is worthwhile to evaluate the effects of these compounds to prevent the occurrence and progression of AP in a mouse model.

*CASC2* is well studied in different cancers in which it acts as a tumor-suppressive lncRNA through regulating the expression of oncogenic microRNAs such as miR‐18a, miR‐21 and miR‐181a and affecting oncogenic pathways such as Wnt/β‐catenin, Ras-Raf-MEK-ERK and JNK signaling pathways 24-29, 43. Recently, *CASC2* is reported to be upregulated in osteoarthritis patients and its overexpression is positively correlated with *IL17* level 23. Overexpression of *CASC2 in vitro* resulted in the upregulation of *IL17* mRNA level 23. Consistent with this result, we also observe that overexpression or knockdown of *CASC2* can change the *IL17* mRNA level. Except for *IL17*, we also found that the expression of another important proinflammatory gene *IL6* is dependent on *CASC2*. Our results for the first time reveal the upstream regulatory mechanism of *CASC2* dysregulation, which will provide a reference for elucidating the mechanism of aberrant expression of *CASC2* in other biological processes. Meanwhile, our results will also benefit to deepen the understanding of regulatory mechanisms of other lncRNAs (e.g., *MALAT1*, *Mirt2* and *ROCK1*) involved in inflammation response. Except for *CASC2*, we will explore the functions of other dysregulated lncRNAs in the pathogenesis of AP in the future.

In summary, our results reveal that DNA methylation and inflammation stimuli coactivate the expression of *CtBPs*. Their encoding proteins associates with PCAF and c-MYC to assemble the CPM complex, which subsequently activates the expression of *CASC2*, *IL6* and *IL17*, aggravating inflammation response and eventually leading to the occurrence of AP. Our results will benefit the research in this field and provide new avenues for the diagnosis and therapy of AP.

## Supplementary Material

Supplementary figures and tables.
