# Supplementary material for: Inflammation and DNA methylation coregulate the CtBP-PCAF-c-MYC transcriptional complex to activate the expression of a long non-coding RNA CASC2 in acute pancreatitis
Source: Int J Biol Sci. 2020 May 18;16(12):2116–30. doi: 10.7150/ijbs.43557 (PMC7294942; doi:10.7150/ijbs.43557)
Supplement: Supplementary file 1 — Supplementary figures and tables. [file ijbsv16p2116s1.pdf]

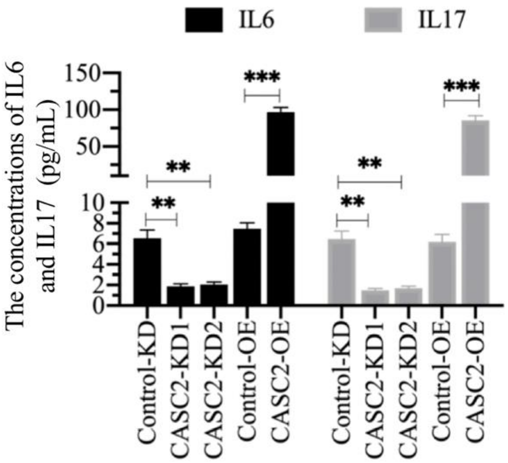

Supplementary Figure 1. The concentrations of IL6 and IL17 in the supernatant of cell culture of CASC2-KD and CASC2-OE cells

The Control-KD, CASC2-KD1, CASC2-KD2, Control-OE and CASC2-OE cells were cultured in DMEM medium for 24 h. Cell cultures were collected and centrifuged, and the supernatant was used to measure the concentrations of IL6 and IL17. $^{**}P < 0.001$ and $^{***}P < 0.001$.

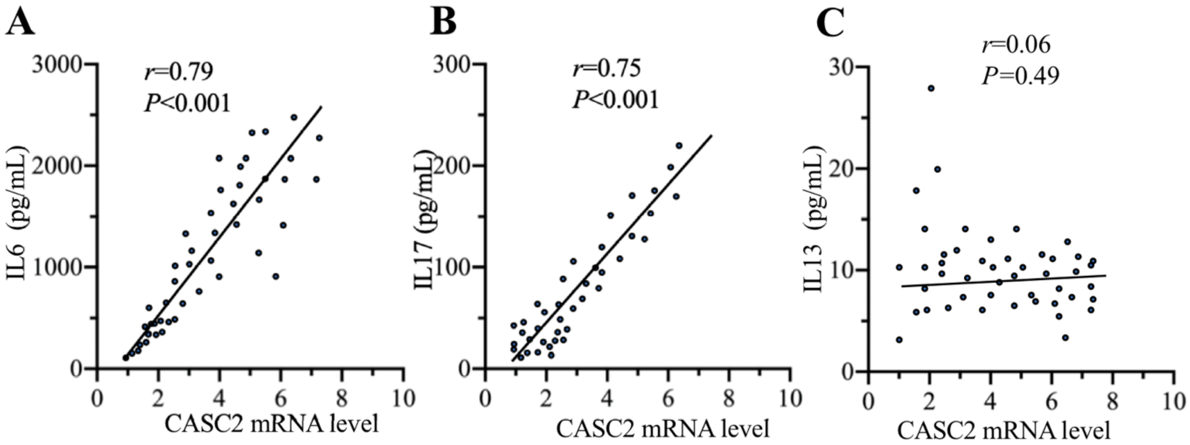

Supplementary Figure 2. IL6 and IL17 were positively correlated with *CASC2* expression

The Pearson correlation coefficient (*r*) and associated probability (*P*) were performed to determine the correlation between IL6 **(A)**, IL17 **(B)** and IL13 **(C)** using the circulating concentrations of cytokines and the relative mRNA level of *CASC2* from the same AP patients.

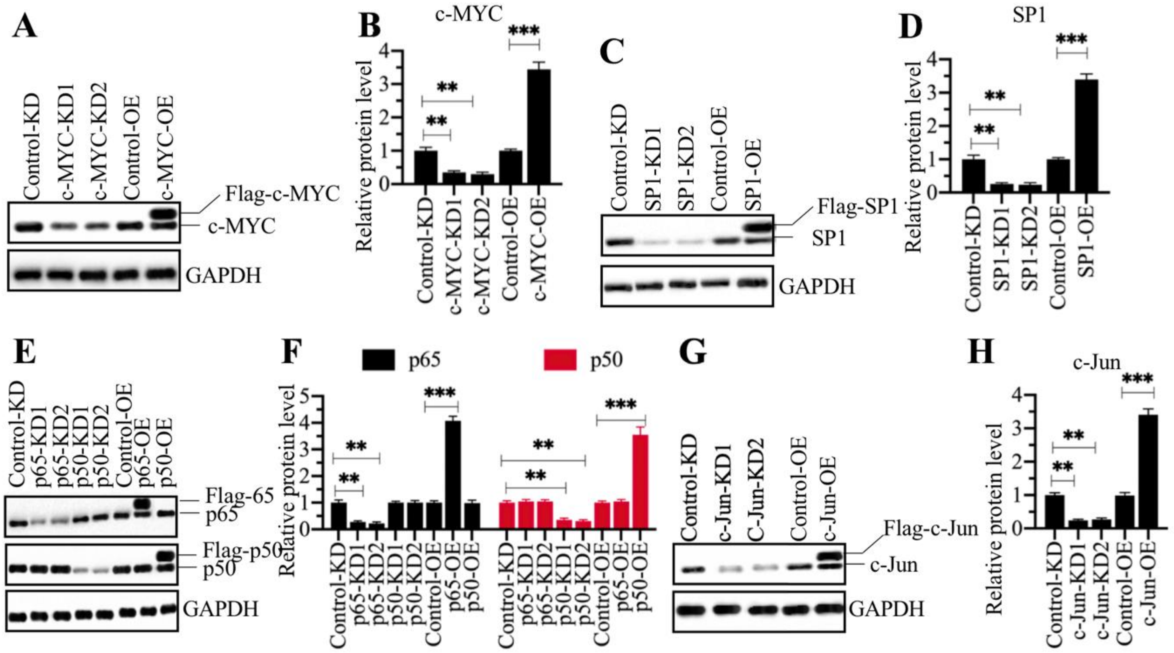

Supplementary Figure 3. The protein levels of transcription factors in their corresponding knockdown and overexpression cell lines

(A) The protein level of c-MYC. Total cell extracts from Control-KD, c-MYC-KD1, c-MYC-KD2, Control-OE, and c-MYC-OE cells were applied to immunoblots to measure c-MYC protein level. **(B)** The relative protein level of c-MYC. The protein signals in (A) were quantified using Image J and normalized to their corresponding GAPDH level. $**P<0.01$ and $***P<0.001$. **(C)** The protein level of SP1. Total cell extracts from Control-KD, SP1-KD1, SP1-KD2, Control-OE, and SP1-OE cells were applied to immunoblots to measure SP1 protein level. **(D)** The relative protein level of SP1. The protein signals in (C) were quantified using Image J and normalized to their corresponding GAPDH level. $**P<0.01$ and $***P<0.001$. **(E)** The protein levels of NF-κB subunits. Total cell extracts from Control-KD, p50-KD1, p50-KD2, p65-KD1, p65-KD2, Control-OE, p50-OE and p65-OE cells were applied to immunoblots to measure the protein levels of p50 and p65. **(F)** The relative protein levels of NF-κB subunits. The protein signals in (E) were quantified using Image J and normalized to their corresponding GAPDH level. $**P<0.01$ and $***P<0.001$. **(G)** The protein level of c-JUN. Total cell extracts from Control-KD, c-JUN-KD1, c-JUN-KD2, Control-OE, and c-JUN-OE cells were applied to immunoblots to measure c-JUN protein level. **(H)** The relative protein level of c-JUN. The

protein signals in (G) were quantified using Image J and normalized to their corresponding GAPDH level. **P<0.01 and ***P<0.001.

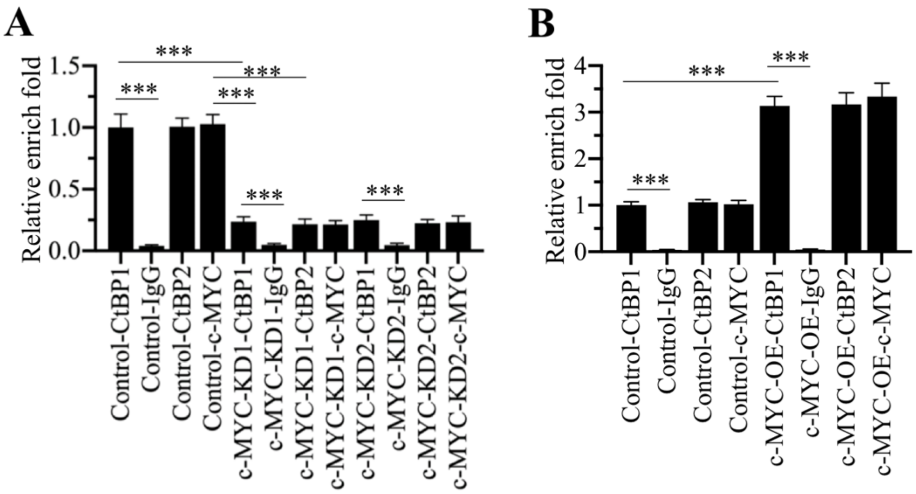

Supplementary Figure 4. The CPM complex specifically bond to the promoter of *CASC2*

(A) Knockdown of *c-MYC* decreased the occupancies of CFM components in the promoter of *CASC2*. The Control-KD, c-MYC-KD1 and c-MYC-KD2 cells were subjected to ChIP assays using IgG, anti-CtBP1, anti-CtBP2 and anti-c-MYC antibodies, respectively. **P < 0.01 and ***P < 0.001. **(B)** Overexpression of *c-MYC* increased the occupancies of CFM components in the promoter of *CASC2*. The Control-OE and c-MYC-OE cells were subjected to ChIP assays using IgG, anti-CtBP1, anti-CtBP2 and anti-c-MYC antibodies, respectively. ***P < 0.001.

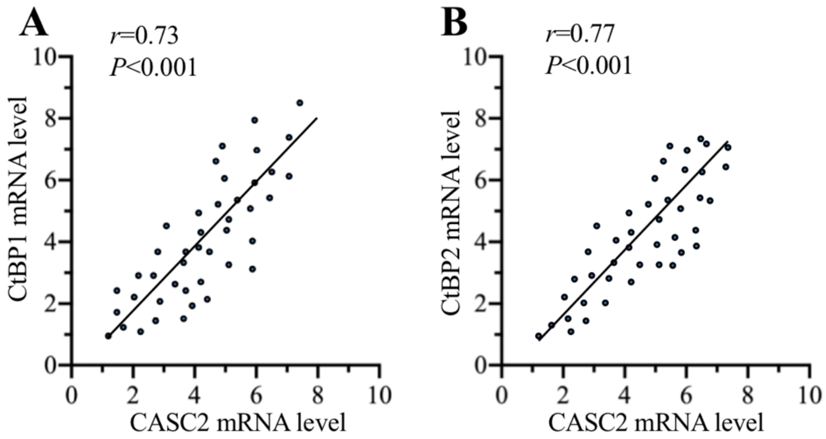

Supplementary Figure 5. CtBP1 and CtBP2 were positively correlated with *CASC2* expression

The Pearson correlation coefficient (*r*) and associated probability (*P*) were performed to determine the correlation between CtBP1 **(A)** and CtBP2 **(B)** using their relative mRNA levels in the same AP patients.

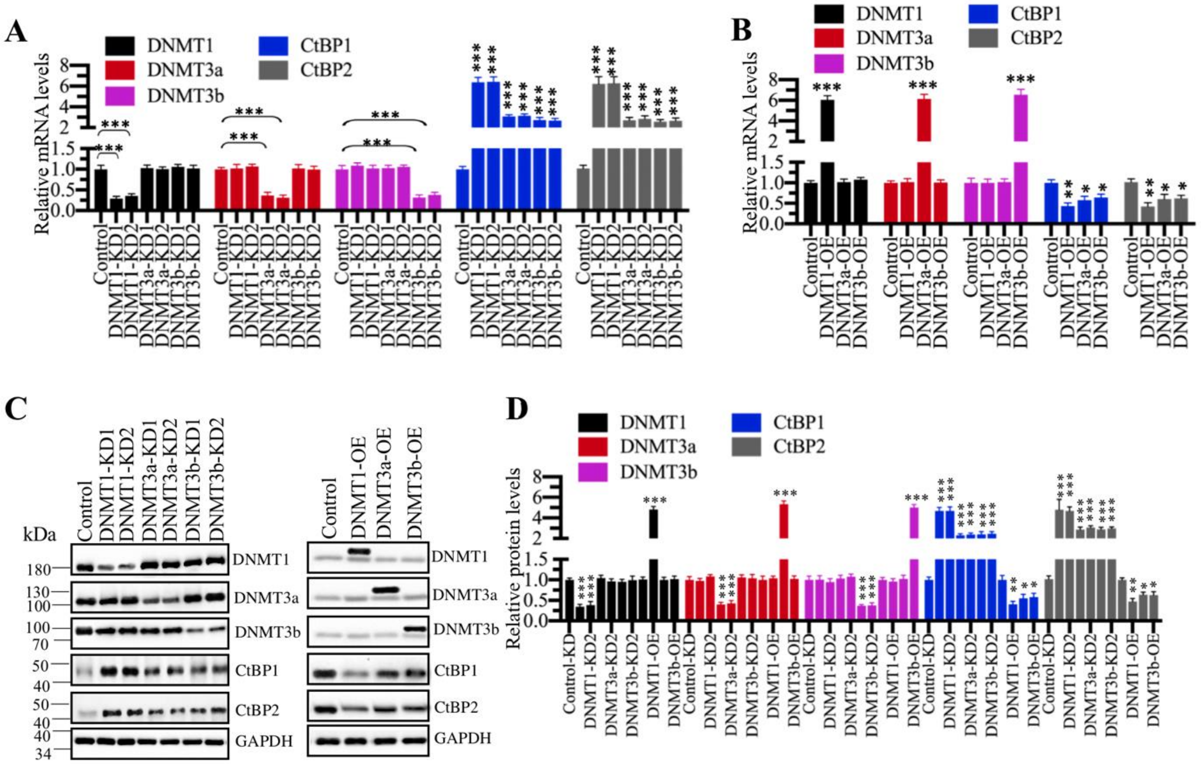

Supplementary Figure 6. Knockdown or overexpression of *DNMTs* changed the expression of *CtBPs*

(A) Knockdown of *DNMTs* increased the expression *of CtBPs*. The Control-KD, DNMT1-KD1, DNMT1-KD2, DNMT3a-KD1, DNMT3a-KD2, DNMT3b-KD1 and DNMT3b-KD2 cells were subjected to RNA isolation, followed by qRT-PCR analyses to measure the mRNA levels of *DNMT1*, *DNMT3a*, *DNMT3b*, *CtBP1* and *CtBP2*. ***$P < 0.001$. **(B)** Overexpression of *DNMTs* decreased the expression *of CtBPs*. The Control-OE, DNMT1-OE, DNMT3a-OE and DNMT3b-OE cells were subjected to RNA isolation, followed by qRT-PCR analyses to measure the mRNA levels of *DNMT1*, *DNMT3a*, *DNMT3b*, *CtBP1* and *CtBP2*. ***$P < 0.001$. **(C)** The effects of knockdown or overexpression of *DNMTs* on CtBP protein levels. The DNMT-KD and DNMT-OE cells used in (A) and (B) were subjected to immunoblots to examine protein levels of DNMT1, DNMT3a, DNMT3b, CtBP1 and CtBP2. GAPDH was used as a loading control. **(D)** The relative protein levels of CtBPs in DNMT-KD and DNMT-OE cells. The protein signal intensity in (C) was quantified and normalized to GAPDH. *$P < 0.05$, **$P < 0.01$ and ***$P < 0.001$.

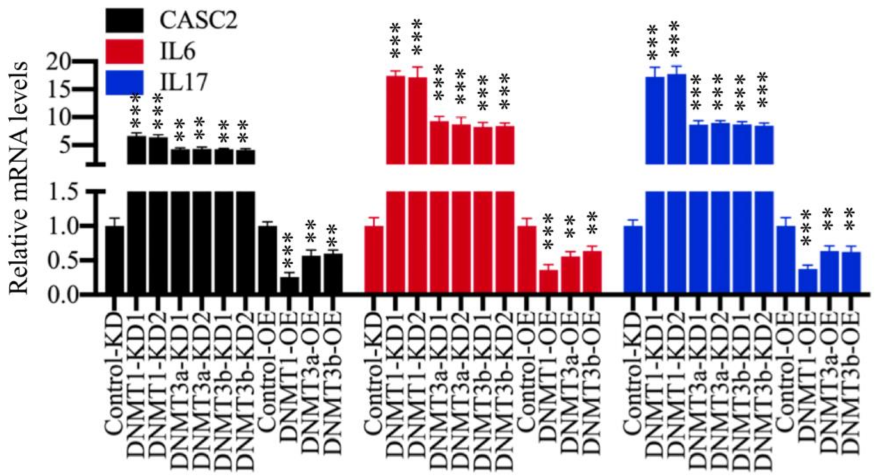

Supplementary Figure 7. The expression of *CASC2*, *IL6* and *IL17* in DNMT-KD and DNMT-OE cells

Total RNA from Control-KD, DNMT1-KD1, DNMT1-KD2, DNMT3a-KD1, DNMT3a-KD2, DNMT3b-KD1, DNMT3b-KD2, Control-OE, DNMT1-OE, DNMT3a-OE and DNMT3b-OE cells were subjected to qRT-PCR analyses to measure the expression of *CASC2*, *IL6* and *IL17*. $^{**}P < 0.01$ and $^{***}P < 0.001$.

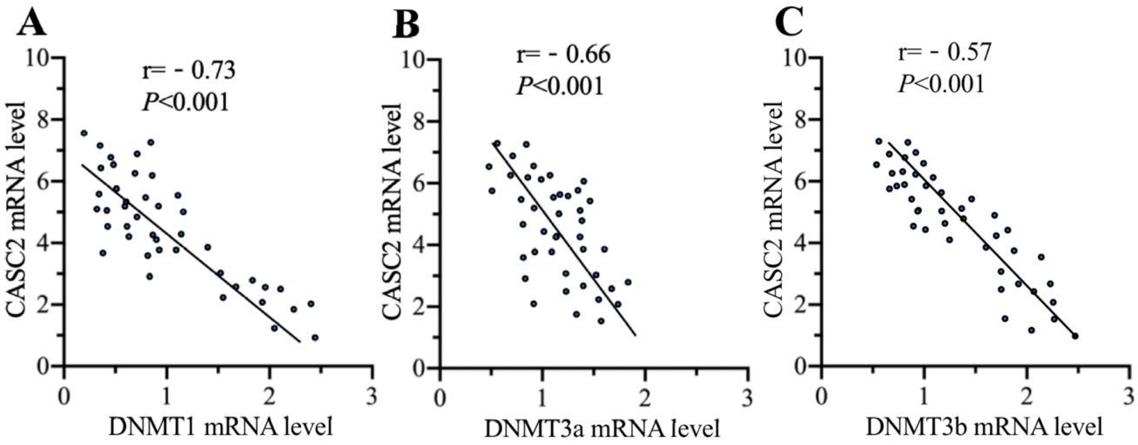

Supplementary Figure 8. The expression of *DNMTs* was negatively correlated with *CASC2* expression

The Pearson correlation coefficient (*r*) and associated probability (*P*) were performed to determine the correlation between DNMT1 **(A)**, DNMT3a **(B)** and DNMT3b **(C)** using their relative mRNA levels in the same AP patients.

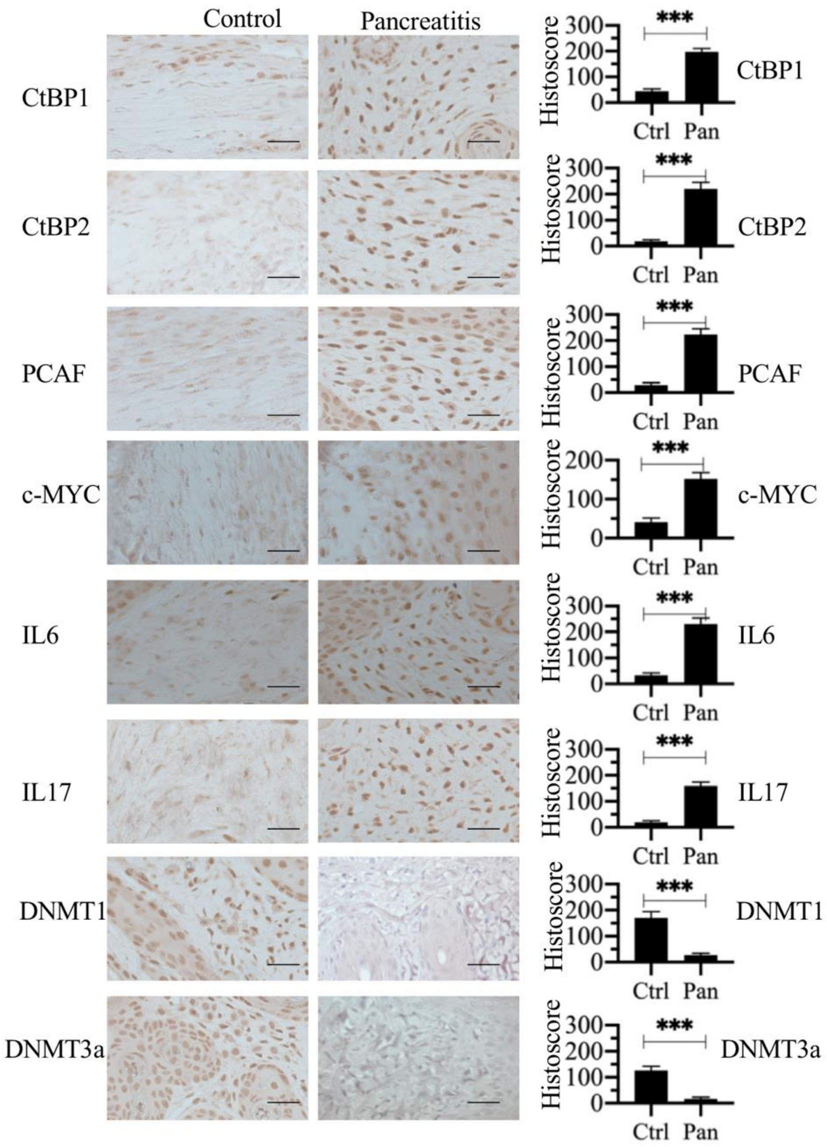

Supplementary Figure 9. The IHC staining results of CPM components and DNMTs in pancreatic tissues

Three-paired pancreatic tissues from controls (stage 0 pancreatic cancer patients) and AP patients were subjected to IHC staining assays using anti-CtBP1, anti-CtBP2, anti-PCAF, anti-c-MYC, anti-IL6, anti-IL17, anti-DNMT1 and anti-DNMT3a, respectively. The representative pictures from the same control and AP patient were shown. Bars=100 μm. Histoscore was calculated by a semi-quantitative assessment of both the intensity of staining and the percentage of positive cells. ***$P < 0.001$.

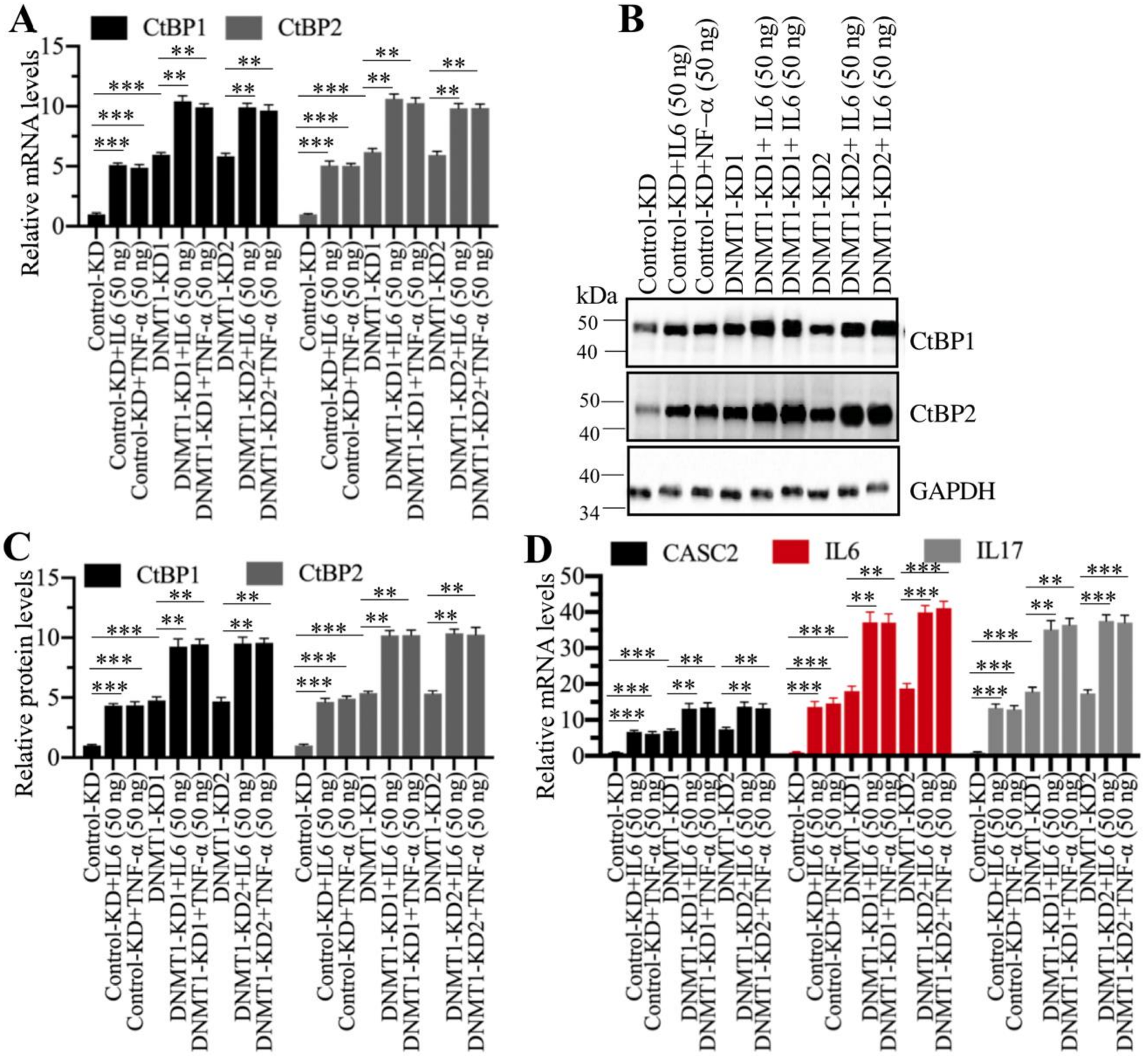

Supplementary Figure 10. Inflammation and DNA methylation coactivated the expression of *CtBPs*

(A) Combined effects of *DNMT1* knockdown and IL6 (or TNF-α) treatment on *CtBP* mRNA levels. The Control-KD and DNMT1-KD cells were treated with or without 50 ng/mL IL6 or TNF-α. The resulting cells were used for RNA isolation, followed by qRT-PCR analyses to determine the mRNA levels of *CtBP1* and *CtBP2*. ****P*<0.001. **(B)** Combined effects of *DNMT1* knockdown and IL6 (or TNF-α) treatment on CtBP protein levels. Cells used in (A) were subjected to immunoblots to examine protein levels of CtBP1 and CtBP2. GAPDH was probed as a loading control. **(C)** The relative protein levels of CtBPs. The protein bands in (B)

were quantified and normalized to GAPDH. ***P < 0.001. **(D)** Combined effects of *DNMT1* knockdown and IL6 (or TNF-α) treatment on the expression of *CASC2*, *IL6* and *IL17*. RNA samples used in (A) were subjected to examine mRNA levels of *CASC2*, *IL6* and *IL17* by qRT-PCR analyses. ***P < 0.001.

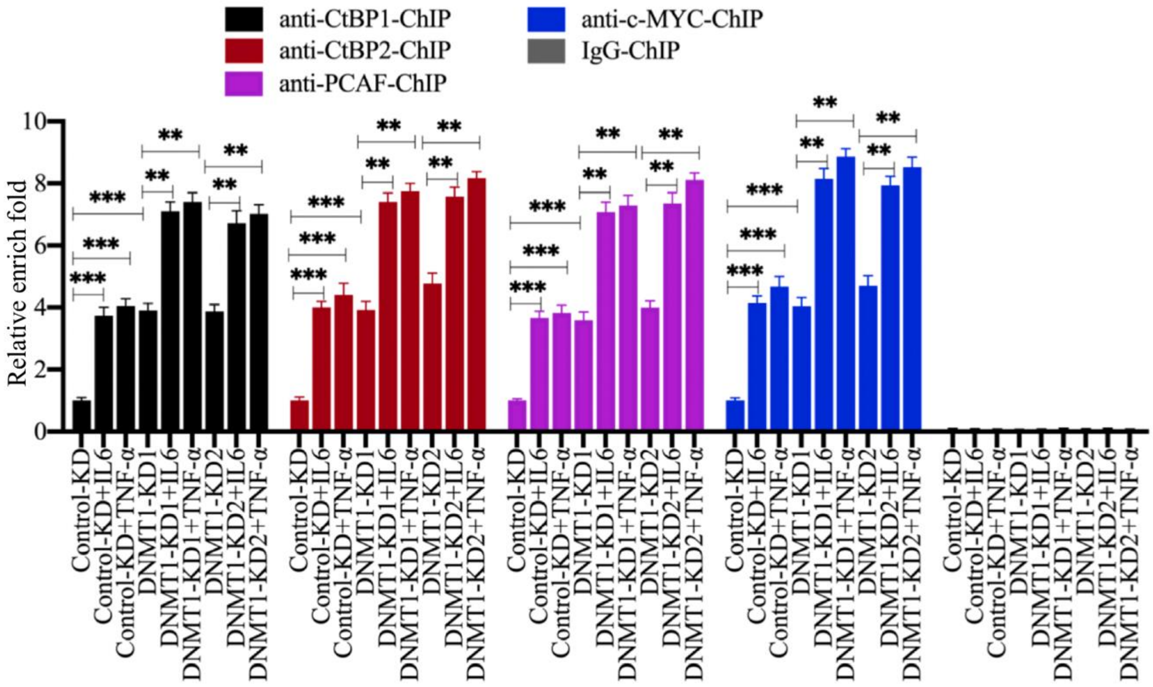

Supplementary Figure 11. Knockdown of *DNMTs* and IL6/TNF-α treatment increased the occupancies of CPM components on the promoter of *CASC2*

The Control-KD, DNMT1-KD1 and DNMT1-KD2 cells were treated with or without 50 ng/mL IL6 and TNF-α, respectively. The treated cells were applied to ChIP assays with anti-CtBP1, anti-CtBP2, anti-PCAF, anti-c-MYC, and IgG (negative control), respectively. The purified DNA was used for qRT-PCR analyses to measure the occupancies of CPM components on the promoter of *CASC2*. $**P < 0.01$ and $***P < 0.001$.

Supplementary Table-1. The basic information of pancreatic cancer patients (Control, n=48) and acute pancreatitis patients (AP, n=48)

Parameters	Control	AP
Mean age	59.3±6.3	53.4±4.5
Gender	30M/18F	33M/15F
Cancer stage	0	NA

F: female; M: male

Supplementary Table-2. Primers used for qRT-PCR analyzes

Gene	Forward Primers	Reverse primers
CtBP1	5’- AGGCGGATAGAGACCACGCCA -3’	5’-CTCTGCAGTGCCAGGGCCACC-3’
CtBP2	5’-TGGAGAAGTTCAAGGCCCTGA-3’	5’-GGCAGATGGTAGAGTCCGCT-3’
DNMT1	5’- AGCAAAGTGAAAGTCATCTACA-3’	5’-GGACTCGAATCTCGCGTAG-3’
DNMT3a	5’-TGAGCGCACAAGAGAGCGGCT-3’	5’-GCACACTCCAGAAAGCAGTT-3’
DNMT3b	5’-GACTCGAAGACGCACAGCTG-3’	5’-GTTGGCAACATCTGAAGCCA-3’
SP1	5’-GTGAGTCTTCCAAGAATCGCAC-3’	5’-CGGTCTGGAACTGTGGGATTA-3’
c-MYC	5’-CAGCAGCAGCAGCAGAGCGAGC-3’	5’-AAGGGTGTGACCGCAACGTAG-3’
IL6	5’- ACCTAGAGTACCTCCAGAACAG-3’	5’- AGATGAGTTGTCATGTCCTGCA-3’
IL17	5’-CTGCTACTGCTGCTGAGCCTGG-3’	5’-TGAGGACCTTTTGGGATTGG-3’
p65	5’-AGTGCGGGACCCATCAGGCA-3’	5’- TCATCCCCACCGAGGCAGCT-3’
p50	5’-AGCTAATCCGCCAAGCAGCT-3’	5’-TTTCAAGTTGGATGCATTGG-3’
c-Jun	5’-GCCTTCGTTAACTGTGTAT-3’	5’- AACACTGGGCAGGATACCC-3’
CDH1	5’- CACCACTGGGCTGGACCGAG-3’	5’-TGGGATTGAAGATCGGAGGA-3’
SOX4	5’-GGAGAAGAAGGTGAAGCGCGT-3’	5’-CTGGGCGCGTCGGGCGAGCA-3’
ALT1	5’-CGCCTTCTCCTTTTCGCAATG-3’	5’-TGTGCAGTTTCAGCAAAGCTC-3’
ADPGK-AS1	5’-GCCGATGTCGACACAAGCG-3’	5’-AGCAAATGTGTTCCCATCCCT-3’
lincIRX5	5’-TCCTGGCTCTCCATGGAGAAG-3’	5’-CCTTCATAGTTGATGACCACCTC-3’
CASC2	5′-GCTGATCAGAGCACATTGGA-3′	5′-ATAAAGGTGGCCACAACTGC-3′
LincROR	5′-CTCCAGCCTAGATGACAGA-3′	5′-CACAGCAGCACTATTCCTAT-3′
MRUL	5′-ACCCACAGACAACTGTGGACCC-3″	5′-GCCGCCCCTATTGTTGCCCA-3′
β-Actin	5’- AGAGCTACGAGCTGCCTGAC-3’	5’- AGCACTGTGTTGGCGTACAG -3’

Supplementary Table-3. Primers used for vector constructions

Gene	Forward Primers	Reverse primers
CtBP1	5’-CGGGATCCATGGGCAGCTCGCACTTGCTCA3’	5’-CGGAATTCCTACAACTGGTCACTGGCGTGGT3’
CtBP2	5’-CGGGATCCATGGCCCTTGTGGATAAGCACAA-3’	5’-CGGAATTCCTATTGCTCGTTGGGGTGCTCTCGA-3’
PCAF	5’-CGGGATCCATGTCCGAGGCTGGCGGGGCC-3’	5’-CGGAATTCTCACTTGTCAATTAATCCAGC-3‘
DNMT1	5’-CGGGATCCATGCCGGCGCGTACCGCCCCA-3’	5’-CGGAATTCCTAGTCCTTAGCAGCTTCCTC-3’
DNMT3a	5’-CGGGATCCATGCCCGCCATGCCCTCCAGCG-3’	5’-CGGAATTCTTACACACACGCAAAATACTCCTTC-3’
DNMT3b	5’-CGGGATCCATGAAGGGAGACACCAGGCATCT-3’	5’-CGGAATTC CTATTCACATGCAAAGTAGTCC-3’
SP1	5’-CGGGATCCATGAGCACCAAGATCACTCCATG-3’	5’-CGGAATTCTCAGAAGCCATTGCCACTGATA-3’
c-MYC	5’-CGGGATCCATGCCCCTCAACGTTAGCTTCA-3’	5’-CGGAATTCTTACGCACAAGAGTTCCGTAGC-3’
p65	5’-CGGGATCCATGGACGAACTGTTCCCCCTC-3’	5’-CGGAATTCTGCTGAGTCAGATCAGCTCCTAA-3’
p50	5’-CGGGATCCATGGCAGAAGATGATCCATATT-3’	5’-CGGAATTCCTAAATTTTGCCTTCTAGAGGTC-3’
c-JUN	5’-CGGGATCCATGACTGCAAAGATGGAAACGAC-3’	5’-CGGAATTCTCAAAATGTTTGCAACTGCTGC-3’

Supplementary Table-4. Primers used for qMSP assays.

Gene	Forward Primers	Reverse primers
CpG1-CtBP1	5'-TTGGTTGAGGGTTTAGTATTGTTAG-3'	5'-AATAATTACATAATTTCAAAAACCAC-3'
CpG2-CtBP1	5'-AGTTTTTGGGTGAGTAGGTTTAGTG-3'	5'-AAATTCAAAACTAAAAAACCCCTTC-3'
CpG1-CtBP2	5'-TATTTGTAGTATAGAGGGTTTTTTTT-3'	5'- AACTCCAACTTCCTCCTAATACC-3'
CpG2-CtBP2	5'- TTTTAAATGGTTTTGAATTAATGAAGG-3'	5'- TCTCAAATCAAAAAACAAAACAATC-3'

Supplementary Table-5. The aberrantly expressed lncRNAs in AP tissues

Ensembl Gene ID	lncRNA name	Average fold change	P Value	Expression
ENSG00000231607	ALT1	-13.4	0.0024	Down
ENSG00000260105	ACO4	-11.1	0.00043	Down
ENSG00000233026	AC026166.2-001	-10.2	0.00041	Down
ENSG00000227033	AC105461.1	-9.3	0.00033	Down
ENSG00000233858	AC026904.1	-8.5	0.00065	Down
ENSG00000267296	CEBPA-AS1	-6.7	0.00099	Down
ENSG00000260898	ADPGK-AS1	-5.9	0.00021	Down
ENSG00000281406	BLACAT1	-5.2	0.00046	Down
ENSG00000280977	lincIRX5	-4.5	0.0013	Down
ENSG00000177640	CASC2	14.8	0.00024	Up
ENSG00000213453	FTH1P3	13.7	0.00022	Up
ENSG00000234741	GAS5	11.3	0.00076	Up
ENSG00000228630	HOTAIR	10.4	0.0083	Up
ENSG00000275874	LINC00162	9.4	0.0024	Up
ENSG00000224259	LINC01133	8.6	0.00016	Up
ENSG00000172965	AWPPH	7.8	0.00054	Up
ENSG00000281183	lncRNA-LET	7.6	0.00025	Up
ENSG00000258609	linc-RoR	6.7	0.00065	Up
ENSG00000246582	LOC389641	5.4	0.00041	Up
ENSG00000225783	Gomafu	5.3	0.00066	Up
ENSG00000135164	MRUL	4.5	0.00084	Up

Supplementary Table-6. The aberrantly expressed genes dependent on *CASC2*

Gene	CASC2-KD1	CASC2-KD2	CASC2-KD3	CASC2-OE1	CASC2-OE2	CASC2-OE3
IL6	-12.1	-9.3	-9.1	12.1	13.5	14.1
IL17	-10.4	-11.1	-8.6	11.1	10.4	12.1
S100A8	-9.3	-8.5	-7.2	9.4	10.1	11.2
S100A9	-8.8	-8.2	-7.1	8.5	7.7	9.3
RUNX1	-6.5	-5.4	-6.6	5.7	4.6	6.3
IDO1	-5.9	-4.5	-5.4	5.1	5.5	5.7
NCF2	-5.4	-4.1	-4.9	4.8	4.2	4.4
PDLIM4	-4.8	-3.6	-4.2	4.5	3.6	2.5
GBP2	-4.1	-3.1	-3.6	3.7	2.9	2.3
SOX4	8.6	7.3	5.3	-6.5	-7.4	-8.3
CDH1	7.2	5.7	4.3	-5.3	-6.9	-6.2
PDZD8	5.4	6.6	3.7	-5.1	-5.4	-5.6
DAND5	4.3	5.3	4.6	-4.8	-4.5	-5.1
PITX3	3.7	4.1	3.5	-4.3	-4.2	-5.0
SYVN1	3.6	4.1	3.5	-4.2	-4.1	-4.7
SLC18A2	3.5	4.0	3.4	-4.1	-4.0	-4.2
CACUL1	3.4	4.0	3.2	-3.9	-3.9	-4.0
PAX5	3.4	3.9	3.1	-3.9	-3.9	-3.7
CSF2	3.1	3.8	3.2	-3.7	-3.8	-3.6
GNB1	3.1	3.6	3.0	-3.6	-3.4	-3.5
SETD2	3.1	3.5	2.9	-3.6	-3.3	-3.3
RTN4R	2.9	3.2	2.8	-3.4	-3.3	-3.2
GSTP1	2.7	3.1	2.6	-3.3	-3.1	-2.9
CBR1	2.6	2.8	2.5	-3.2	-3.0	-2.7
LMO2	2.4	2.5	2.4	-2.8	-2.9	-2.2

Supplementary Table-7. **The c-MYC-associated proteins identified by mass spectrometry**

Protein	Protein description	Molecular weight (kDa)	MASCOT scores
c-MYC	MYC Proto-Oncogene, BHLH Transcription Factor	49	1053
β-Actin	Actin Beta	55	2021
CtBP1	C-Terminal Binding Protein 1	49	967
CtBP2	C-Terminal Binding Protein 2	49	954
PCAF	P300/CBP-Associated Factor	93	933
MAX	MYC Associated Factor X	48	921
MED1	Mediator Complex Subunit 1	168	843
SUPT3H	Suppressor Of Ty 3 Homolog	36	811
BIN1	Bridging Integrator 1	86	765
MED16	Mediator Complex Subunit 16	65	712
ZEB1	Zinc Finger E-Box Binding Homeobox 1	124	687
HIPK2	Homeodomain Interacting Protein kinase 2	131	667
TADA3	Transcriptional Adaptor 3	49	645
USP7	Ubiquitin Specific Peptidase 7	128	632
TAF6	TATA-Box Binding Protein Associated Factor 6	73	612
MYOD1	Myogenic Differentiation 1	35	589
H3C1	H3 Clustered Histone 1	15	567
GATA1	GATA Binding Protein 1	43	562
USP22	biquitin Specific Peptidase 22	60	532
GPS2	G Protein Pathway Suppressor 2	37	511
NRIP1	Nuclear Receptor Interacting Protein 1	127	508
TRNT1	TRNA Nucleotidyl Transferase 1	50	495
NOD2	Nucleotide Binding Oligomerization Domain Containing 2	115	477
TLE3	Transducin-Like Enhancer Protein 3	83	464
SEH1L	SEH1 Like Nucleoporin	40	453
CARHSP1	Calcium-regulated Heat Stable Protein 1	16	442
CTRL1	Chymotrypsin-like Protease	28	434
PSMB1	Proteasome subunit Beta 1	26	436
RPL19	Ribosomal Protein L19	23	425
DAZAP1	DAZ Associated Protein 1	43	411
MIDN	Midnolin	49	367
TAF5	TATA-Box Binding Protein Associated Factor 5	87	352
DNM2	Dynamin 2	98	314
TAF3	TATA-Box Binding Protein Associated Factor 3	103	305
BIN2	Bridging Integrator 2	62	299
SBF2	SET Binding Factor 2	208	254
